# Cooperative ordering of treadmilling filaments in cytoskeletal networks of FtsZ and its crosslinker ZapA

**DOI:** 10.1038/s41467-019-13702-4

**Published:** 2019-12-17

**Authors:** Paulo Caldas, Mar López-Pelegrín, Daniel J. G. Pearce, Nazmi Burak Budanur, Jan Brugués, Martin Loose

**Affiliations:** 1 Institute of Science and Technology Austria, 3400 Klosterneuburg, Austria; 2 School of Chemistry and Biochemistry, University of Geneva, 1211 Geneva, Switzerland; 3 Max Planck Institute of Molecular Cell Biology and Genetics, 01307 Dresden, Germany; 4 Max Planck Institute for the Physics of Complex Systems, 01187 Dresden, Germany; 5 Centre for Systems Biology Dresden, 01307 Dresden, Germany; 6 Cluster of Excellence Physics of Life, TU Dresden, 01062 Dresden, Germany

**Keywords:** Biochemistry, Biophysics, Single-molecule biophysics, Cytoskeleton, Biological physics

## Abstract

During bacterial cell division, the tubulin-homolog FtsZ forms a ring-like structure at the center of the cell. This Z-ring not only organizes the division machinery, but treadmilling of FtsZ filaments was also found to play a key role in distributing proteins at the division site. What regulates the architecture, dynamics and stability of the Z-ring is currently unknown, but FtsZ-associated proteins are known to play an important role. Here, using an in vitro reconstitution approach, we studied how the well-conserved protein ZapA affects FtsZ treadmilling and filament organization into large-scale patterns. Using high-resolution fluorescence microscopy and quantitative image analysis, we found that ZapA cooperatively increases the spatial order of the filament network, but binds only transiently to FtsZ filaments and has no effect on filament length and treadmilling velocity. Together, our data provides a model for how FtsZ-associated proteins can increase the precision and stability of the bacterial cell division machinery in a switch-like manner.

## Abstract (web summary)

The Z-ring, constituted of the tubulin homolog FtsZ protein, plays an essential role for bacterial cell division. Here the authors use an in vitro reconstitution approach to determine how the regulatory protein ZapA affects FtsZ treadmilling and filament organization into large-scale patterns.

## Introduction

For cytokinesis, bacteria need to build two new cell poles at the division site. This process is initiated and orchestrated by a dynamic ring of FtsZ filaments. This Z-ring forms at mid-cell early during the cell cycle, where it persists during the inward growth of the cell septum until it disassembles just before division is completed^1–4^. Recent studies have shown that the Z-ring not only defines the location of divisome assembly, but that FtsZ treadmilling also drives the movement of peptidoglycan synthases around the cell diameter, which in turn controls remodeling of the cell wall at the division site^5,6^. Due to the important role of FtsZ polymerization dynamics for septal cell wall synthesis, the spatial and temporal organization of filaments within the Z-ring needs to be tightly controlled. Indeed, bacteria contain various proteins that were found to directly bind to FtsZ and to be critical for the positioning and stability of the division machinery^7–9^. For example, mutants of *Escherichia coli* lacking one of the FtsZ-associated proteins ZapA, ZapB, ZapC or ZapD are usually longer than wild-type cells with a more heterogeneous cell length distribution. Furthermore, they show mislocalized or misaligned Z-rings and skewed division septae, with their corresponding phenotype becoming even more severe in cells missing more than one Zap protein^8–11^. Despite their important role for cell division, the molecular mechanism for how these proteins influence the dynamic behavior of FtsZ filaments and their organization into the Z-ring is currently not known.

The best characterized FtsZ-associated protein is ZapA, which is widely conserved across Gram-positive and Gram-negative bacteria. Previous in vitro experiments using only purified FtsZ and ZapA showed that ZapA promotes the formation of stable FtsZ filament bundles when incubated together in solution. A crosslinking activity of ZapA is also consistent with its molecular structure, as it forms a pseudosymmetric tetramer with two distal pairs of FtsZ-binding domains oligomerized via a central four-helix bundle^12–14^. The symmetry of the ZapA tetramer appears to be compatible with an antiparallel orientation of filaments within FtsZ bundles. At the same time, ZapA was found to reduce the GTPase activity of FtsZ suggesting that it can slow down FtsZ polymerization dynamics^15–17^, even though this effect depends on buffer conditions^14,18^. Together, these findings have led to a model where ZapA stabilizes the Z-ring by promoting antiparallel bundling of filaments and reducing polymer turnover^12,16^. However, a direct demonstration of these effects has not yet been possible and it is unclear how decreased filament turnover is compatible with the function of treadmilling FtsZ filaments to homogeneously distribute wall synthases at the division site.

Here, we applied an in vitro reconstitution approach to determine how ZapA affects FtsZ polymerization and large-scale organization of membrane-bound FtsZ filaments into cytoskeletal patterns. For our experiments, we used the purified proteins FtsZ, ZapA and FtsA, the natural membrane anchor of FtsZ, as well as supported lipid bilayers to mimic the cytoplasmic membrane. This approach allowed us to study dynamic interactions between the proteins on a membrane surface mimicking the physiological conditions in the living cell. In addition, by using total internal reflection fluorescence (TIRF) microscopy, we benefit from higher spatiotemporal resolution than in living cells to simultaneously visualize the self-organization of FtsZ and ZapA in vitro. With the help of novel image analysis tools, we then quantified the influence of ZapA on FtsZ on three different spatial scales: First, we analyzed the large-scale organization of the membrane-bound filament network; next, we studied the underlying polymerization dynamics, and finally, we quantified the behavior of single molecules.

Our experiments and analysis show that ZapA acts on FtsZ filaments in a highly cooperative manner: at low ZapA concentrations, the FtsZ filament network shows low spatial order with fast reorganization dynamics, whereas the system switches to a persistent state of high spatial order and slow reorganization dynamics at high concentrations of ZapA. Strikingly, our data shows that despite its pronounced effect on the large-scale organization of the filament network, binding of ZapA to FtsZ filaments is weak and transient, and does not change filament length and orientation. Furthermore, even though the reorganization of the network is slowed down significantly, ZapA leaves the treadmilling rate of FtsZ unchanged.

In summary, our findings provide a mechanism for a how ZapA spatially confines FtsZ treadmilling trajectories to increase the precision of Z-ring assembly. Furthermore, the observed ultrasensitive response of FtsZ to ZapA can contribute to the directional maturation of the cell division machinery to recruit additional components of the cell division machinery to mid-cell. Together, our findings shed new light on the self-organizing properties of the bacterial cytoskeleton.

## Results

### ZapA crosslinks membrane-bound FtsZ filaments

**Fig. 1:**
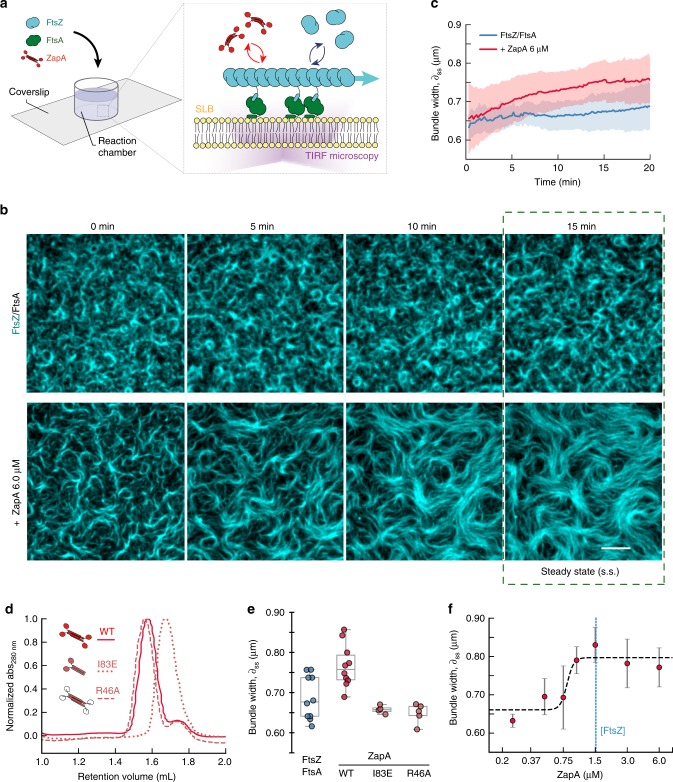
ZapA increases the width of membrane-bound FtsZ filament bundles. **a** Illustration of the experimental assay based on a supported lipid bilayer, purified proteins and TIRF microscopy. **b** Snapshots of FtsZ pattern emerging from its interaction with FtsA alone (top row) and in the presence of 6 μM ZapA (lower row). Concentrations of FtsZ (30% Cy5-labelled) and FtsA were kept constant for all experiments (1.5 μM and 0.5 μM, respectively). Scale bars, 5 μm. **c** Estimated mean bundle width, *δ*, over time for FtsZ/FtsA filament pattern (blue) and with 6 μM ZapA (red). Curves depict the mean and the standard deviation (std) as shaded error bands of independent experiments (FtsZ, *n* = 10, ZapA, *n* = 10). **d** Size-exclusion chromatography results for ZapA wild type (WT) and mutants (ZapA I83E, ZapA R46A). **e** Mean bundle width, *δ*_*ss*_, of membrane-bound FtsZ filaments is increased in the presence of 6 μM ZapA (red) (two-tailed *t* test with *p* = 0.004) while it was unchanged in the presence of ZapA I83E and ZapA R46A (light red) (*p* > 0.05). **f** Change in *δ*_*ss*_ with varying concentrations of ZapA. Data shown (steady state) corresponds to the average bundle width measured between *t* = 15 min and *t* = 20 min (mean ± std). Black line corresponds to Hill equation fit with *n*_H_ = 13.86 and EC_50_ = 0.81 μM with 90% confidence intervals of [13.86–94.68] and [0.71, 0.98], respectively, obtained from bootstrapping distributions. Source data available at Figshare [10.6084/m9.figshare.10347062.v1].

To study the effect of ZapA on the architecture and dynamics of the FtsZ filament network, we took advantage of an in vitro system based on supported lipid bilayers and purified proteins (Fig. 1a). As reported previously, FtsZ and FtsA form treadmilling filaments on supported membranes when incubated in the presence of ATP and GTP and at a concentration ratio similar to that found in the living cell (FtsZ: FtsA = 1.5 μM: 0.5 μM). These filaments further self-organize into dynamic networks of curved filament bundles^19^ (Fig. 1b, top row; Supplementary Movie 1, left). Next, we performed this experiment in the presence of defined concentrations of ZapA. We first used a concentration about four times higher than in vivo (6 μM, ref. ^15^), and found that FtsZ filaments organized into a network of more aligned and straightened filament bundles (Fig. 1b, lower row; Supplementary Movie 1, right). This effect became more pronounced over time until the system assembled into a stable steady state after about 15 min, with an architecture that strongly differed from the pattern found in the absence of ZapA (Fig. 1b, top row). Following these initial observations, we then used automated image analysis methods to quantify the influence of ZapA on the organization of membrane-bound FtsZ filaments.

As ZapA was previously found to promote lateral interactions between FtsZ protofilaments and the formation of filament bundles when incubated together in solution^14,16,17^, we first quantified the apparent width of filament structures on the membrane. For this aim, we used an image segmentation approach based on the fluorescence intensity followed by distance mapping (Supplementary Fig. 1a, b; methods for details). For bundles of membrane-bound FtsZ filaments in the absence of ZapA, our analysis found a mean width of *δ*_*t* *=* *0*_ = 0.63 ± 0.03 μm, which stayed roughly constant over time (*δ*_*ss*_ = 0.70 ± 0.05 μm, *n* = 10; Fig. 1c, blue). In contrast, in the presence of 6.0 μM ZapA, the FtsZ bundle width continuously increased until it plateaued at a slightly higher value of *δ*_*ss*_ = 0.77 ± 0.05 μm (*n* = 10, Fig. 1c, red) at steady state (*t* > 15 min).

As a control, we prepared ZapA R46A, a mutant of ZapA with an inactive FtsZ-binding site (Fig. 1d). As expected, adding this mutant up to a concentration of 6 μM did not change the FtsZ filament pattern and bundles had the same average width as in the absence of ZapA (Fig. 1e, two-tailed t test with *p* = 0.080; Supplementary Fig. 1c, d). To confirm that only tetrameric ZapA with two pairs of FtsZ-binding sites has this effect on FtsZ filaments, we prepared ZapA I83E, a mutant where the dimer-dimer interaction is disrupted^17^ (Fig. 1d). Even though the presence of ZapA I83E slightly changed the appearance of FtsZ filaments (Supplementary Fig. 1d, top row), we did not observe a significant increase of bundle thickness as found for the wild-type protein (Fig. 1e; two-tailed t test with *p* = 0.131; Supplementary Table 1). Accordingly, and in agreement with previous observations^17^, tetramerization of ZapA is essential for its function.

Next, we investigated how varying concentrations of wild-type ZapA affect the FtsZ filament pattern (Fig. 1f and Supplementary Fig. 1e, f). Up to a concentration of ZapA = 0.75 μM, the observed bundle width at steady state was below 0.7 μm and did not change significantly. However, we observed a rapid shift to structures thicker than 0.75 μm at ZapA concentrations higher than 1.0 μM. This value peaked at ZapA = 1.5 μM before it slightly decreased. Interestingly, the observed plateau point occurs at an equimolar stoichiometry of FtsZ and ZapA, matching the concentration ratio found in vivo^14^. These kind of switch-like transitions above a critical threshold are common in biology and are often analyzed by fitting a Hill equation. This analysis confirmed a cooperative transition of the system towards thicker FtsZ bundles with a Hill coefficient much larger than 1 and EC_50_(ZapA) = 0.81 μM (90% confidence interval (CI): 0.71–0.98) (Supplementary Table 2). To summarize, we found that ZapA is able to increase the apparent width of FtsZ filament bundles, even when they are recruited to the membrane by FtsA. Furthermore, having the capability to define the protein concentrations in our in vitro experiments revealed that ZapA acts on FtsZ in a highly cooperative manner.

### ZapA changes the architecture of FtsZ filament networks

**Fig. 2:**
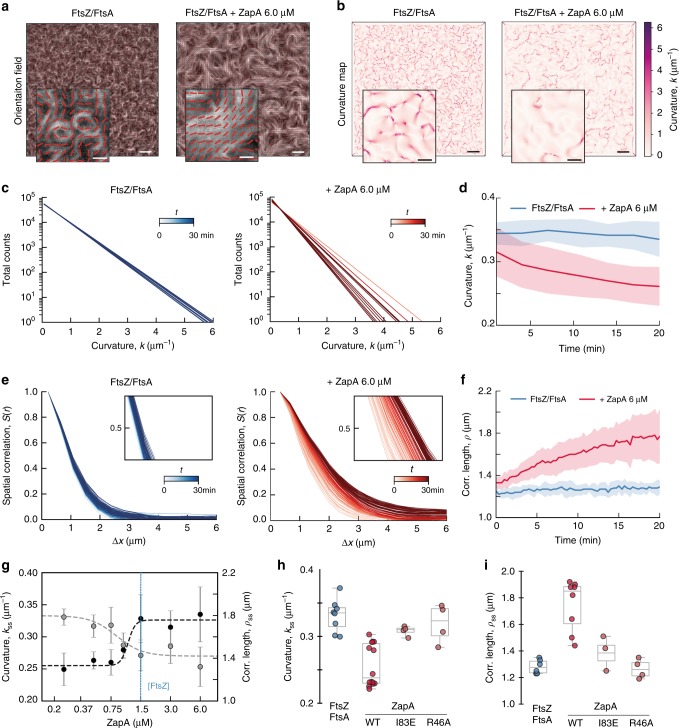
ZapA changes the architecture of the FtsZ filament network. **a** Orientation fields (red lines) for FtsZ filament network without (left) or with 6.0 μM ZapA (right) overlaying corresponding fluorescence micrographs. **b** Curvature maps of orientation fields; white areas correspond to areas with straight filament bundles, while the purple areas corresponds to the degree of curvature. Scale bars: large image, 5 μm; small image, 2 μm. **c** Time-dependent decrease of curvature for FtsZ filaments alone (left, blue gradient, light to dark) and with 6 μM ZapA (right, red gradient, light to dark). **d** The mean curvature, *k*, at each time point was given by the characteristic length scale of an exponential fit to the curvature distributions. Curves depict the mean and std as shaded error bands of independent experiments (FtsZ, *n* = 10, ZapA, *n* = 10). **e** Typical spatial correlation function, S(*r*), for an FtsZ filament network without (left, blue gradient, light to dark) or with 6 μM ZapA (right, red gradient, light to dark). For FtsZ/FtsA the curves overlapped in time while ZapA promoted a shift to higher correlation lengths. **f** Correlation length, *ρ*, was given by the intersection line where S(*r*) = 0.5 (insets in **e**) and shows that *ρ* remained constant for FtsZ/FtsA (blue) over time, but continuously increased with 6 μM ZapA (red). Curves show the mean and std as shaded error bands of independent experiments (FtsZ, *n* = 6, ZapA, n = 8). **g** *k* (gray) and *ρ* (black) with varying concentrations of ZapA. ZapA induced a switch-like behavior for *k*_*ss*_ and *ρ*_ss_ (*n*_H_ = 3.28 and EC50 = 0.84 μM; *n*_H_ = 12.04 and EC50 = 1.05, respectively) with a saturation point close to equimolar concentrations with FtsZ. Circles depict the mean and std of independent experiments (FtsZ, *n* = 6, ZapA, *n* = 8). The 90% confidence intervals for *n*_H_ and EC50 obtained from boostrapping are shown in Supplementary Table 2. **h**, **i** Mean values for ZapA I83E and R46A (light red) shows different behavior compared to wildtype ZapA (red). Source data available at Figshare [10.6084/m9.figshare.10347062.v1].

Bundling of FtsZ filaments does not fully characterize the influence of ZapA, as we also observed a dramatic shift in the overall architecture of the filament network. For example, without ZapA, the filament bundles were highly curved, often forming ring-like structures on the membrane. In contrast, the presence of ZapA gave rise to a pattern of straighter filament bundles, where ring-like structures were missing (Fig. 1b). To better characterize the biochemical activity of ZapA and its influence on FtsZ filament organization, we established a method to obtain a mathematical representation of the filament network (Fig. 2a). Our approach is based on the computation of structure tensors. This involves dividing the image into small regions, each roughly the size of the feature we aim to quantify, in our case 4 pixels corresponding to 432 nm. In each region we calculate a unitary vector describing the average orientation of the gradient of the local gray scale (methods for details). The result is a vector field representing the orientation of the filaments at each point in the image, which can be generated for each frame of a movie. These time-dependent orientation fields can then be analyzed to calculate two independent parameters: the curvature, *k* (μm^−1^) (Fig. 2b–d, g) and mean correlation length, *ρ* (μm) (Fig. 2e-g).

From our curvature analysis, we obtained a heatmap describing the magnitude of the local curvature at each x and y coordinate from the orientation field at each time point (Fig. 2b and Supplementary Fig. 2a). The filament curvatures were shown to be exponentially distributed (Fig. 2c and Supplementary Fig. 2b). The mean curvature of the filament network at each time point, *k*(*t*), was then given by the characteristic scale of an exponential fit to the curvature distribution (Fig. 2c and Supplementary Fig. 2a). In the absence of ZapA, FtsZ filaments showed a constant mean curvature of *k*_*ss*_ = 0.33 ± 0.06 μm^−1^ (Fig. 2d, blue, *n* = 10). In contrast, in the presence of 6 μM ZapA, *k*(*t*) decreased over time by about 20% with a plateau at *k*_*ss*_ = 0.25 ± 0.03 μm^−1^ (Fig. 2d, red, *n* = 10), confirming that the presence of ZapA results in straighter bundles of FtsZ on the membrane.

Next, we used the orientation field to calculate the spatial order of the filament network, which is a measure for how the orientations of the filaments co-vary with one another across the observed membrane area. Specifically, we used the correlation function, *S*(*r*), which represents the average alignment between two filaments at a given distance, *r* (methods for details). At a given time, *S*(*r*) showed a decreasing correlation of alignment with increasing distance (Fig. 2e). In the absence of ZapA, the correlation curves obtained for different time points overlapped with each other (Fig. 2e, left; light to dark blue gradient), showing that spatial order stayed constant during the experiment. The presence of ZapA increases the correlation length, an effect that becomes more pronounced with time, indicating that ZapA increases the spatial order of the system (Fig. 2e, right; light to dark red gradient). We quantified this time-dependent increase in spatial order by defining a characteristic correlation length, *ρ*(*t*), which corresponds to the distance at which *S*(*r*) = 0.5 (Fig. 2e insets and Fig. 2f). We found that the presence of ZapA led to a continuous increase in spatial order from *ρ*(*t* = 0) = 0.97 ± 0.06 μm to *ρ*_*ss*_ = 1.81 ± 0.26 μm at steady state (Fig. 2f, red, *n* = 8), while *ρ*_*t*_ stayed constant for FtsZ and FtsA alone (Fig. 2f, blue, *n* = 6). Both values are similar to the dimensions of the *E. coli* cell, consistent with the spatial scale at which the Z-ring is operating in vivo.

Next, we measured how increasing concentrations of ZapA affect the curvature and correlation length at steady state, *k*_ss_ and *ρ*_*ss*_ (Fig. 2g, Supplementary Fig. 2b, c). Similar as for the bundle width (Fig. 1e), we found a switch-like transition between two states with Hill coefficients of *n*_H_ = 3.28 and *n*_H_ = 12.04, along with a EC_50_ = 0.84 μM and EC_50_ = 1.05 μM for *k*_*ss*_ and *ρ*_*ss*_, respectively (see Supplementary Table 2 for 90% confidence intervals). Furthermore, neither ZapA R46A nor I83E had the same effect on the FtsZ filament network (Fig. 2h, i; *p* > 0.05, Supplementary Table 1). These results show that the system exists in two states: a state of high curvature and low spatial order, at ZapA concentrations below 0.8 µM, and a state of high spatial order and low curvature at higher concentrations.

### ZapA stabilizes the membrane-bound FtsZ filament network

**Fig. 3:**
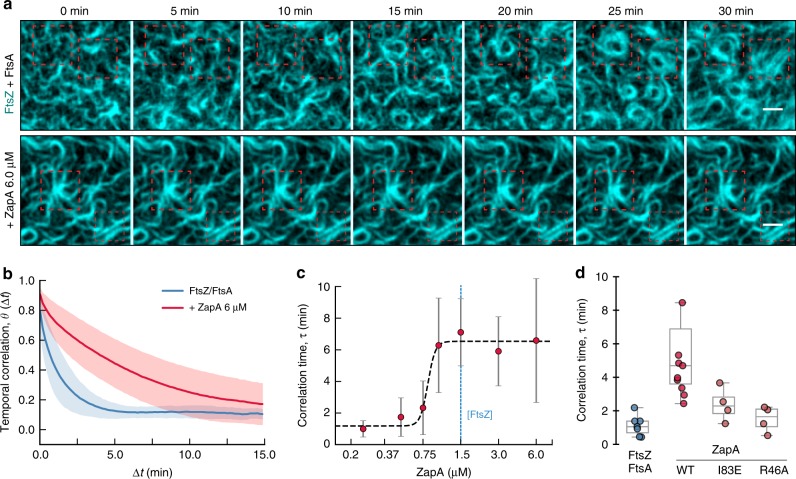
ZapA stabilizes FtsZ filament bundles. **a** In the absence of ZapA (upper panel), the FtsZ filament pattern constantly reorganized and ring-like structures formed only transiently (red dashed squares). In contrast, in the presence of ZapA (lower panel) FtsZ assemblies were much more persistent. Scale bars, 2 μm. **b** The temporal autocorrelation function of the FtsZ filament network decayed quickly without ZapA (blue), but slowly in the presence of 6.0 μM (red). Each curve represents the mean ± std (error bands) of independent experiments (FtsZ, *n* = 7, ZapA, *n* = 9). **c** The characteristic correlation time, *τ*, as a function of ZapA concentration. The autocorrelation curves were fitted to a mono-exponential decay and *τ* was given by the half-time of each decay. Once again, ZapA induced a switch-like behavior showing a hill coefficient » 4 and a EC50 = 1.05 (Supplementary Table 2). Data shown corresponds do the average *τ* from individual experiments (mean ± std). **d** ZapA I83E and R46A (light red) did not change the persistence of FtsZ filament bundles (*p* > 0.05, Supplementary Table 1). Source data available at Figshare [10.6084/m9.figshare.10347062.v1].

Z-rings in cells lacking ZapA not only show loose filament arrangement, but are also highly dynamic, transitioning back and forth between multiple locations in the cell^9^. In contrast, the Z-ring in wildtype cells usually persists at the same position during the cell cycle. Consistent with these observations, we realized that FtsZ filament bundles continuously reorganized in the absence of ZapA, but appeared much more static when ZapA was present. To quantify the rate of reorganization, we performed temporal autocorrelation analysis. This method measures the similarity of fluorescence images as a function of a time lag Δt between different frames (Fig. 3a; see methods for details). We found that the corresponding autocorrelation function, ϴ(Δt), rapidly decayed in the absence of ZapA, consistent with a rapidly rearranging pattern (Fig. 3b, blue curve). In the presence of 6 μM of ZapA, however, this decay was about 4-fold slower (Fig. 3b, red curve). We defined the characteristic correlation time, *τ*, for varying concentrations of ZapA by the halftime of an exponential fit to the correlation curves. Similar to bundle width, curvature and correlation length, *τ* displayed a two-state behavior: below a critical concentration of ZapA the pattern showed fast reorganization and consequently short correlation times, but the system switched to slow reorganization and long correlation times at saturating ZapA concentrations with a Hill coefficient of 14.55 (90% CI: 9.22–69.27) and EC_50_(ZapA) = 0.82 (90% CI: 0.76–0.87) (Fig. 3c, Supplementary Table 2). ZapA R46A, which does not bind to FtsZ, did not affect reorganization dynamics (Fig. 3d; two-tailed t test with *p* = 0.427), while ZapA I83E increased slightly, but not-significantly the correlation time (Fig. 3d; two-tailed *t* test with *p* = 0.084). This shows that tetrameric ZapA not only affects the architecture of the membrane-bound filament network, but also its reorganization dynamics in a highly cooperative manner.

Together, our in vitro experiments and quantitative image analysis identified an ultrasensitive switch in filament architecture and reorganization dynamics depending on the concentration of ZapA. In all instances, this switch happens at ZapA concentrations between 0.75 and 1.0 μM, before the system saturates at ZapA concentrations equimolar to FtsZ at 1.5 μM (Supplementary Table 3). Interestingly, this correlation is consistent with ZapA tetramers having four FtsZ-binding sites and with their concentration ratio found in vivo^12,14^.

### ZapA does not change FtsZ filament orientation

So far, our experiments revealed that ZapA significantly slows down reorganization of the dynamic FtsZ filament network and increases its spatial order. We next considered how ZapA affects the FtsZ network architecture on a filament level, i.e. how does ZapA affect filament orientation, length and treadmilling velocity? The observed directional treadmilling of FtsZ filament bundles in vivo and in vitro is generally compatible with a polar orientation of filaments. However, based on its molecular structure, ZapA was suggested to promote their antiparallel alignment^12^. Accordingly, we wondered how the presence of ZapA changes the relative orientation of FtsZ filaments in our assay.

**Fig. 4:**
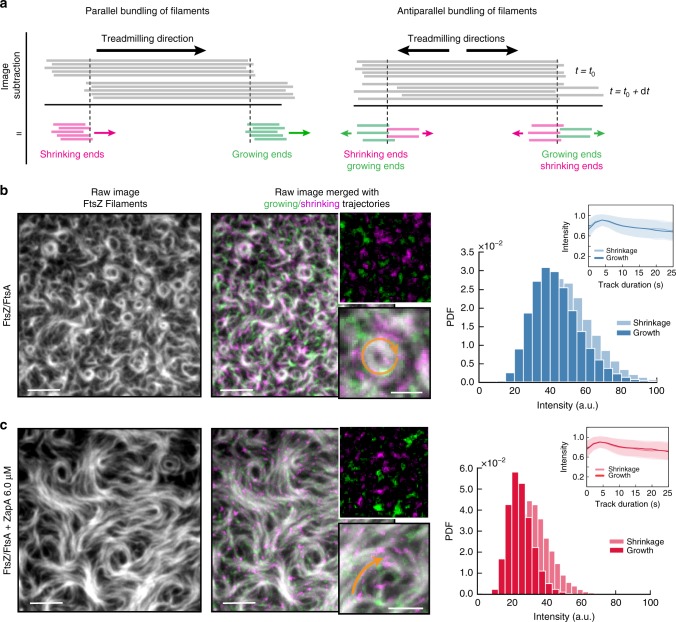
ZapA does not change orientation of FtsZ filaments. **a** Illustration of image subtraction step assuming parallel (left panel) or antiparallel filament bundles (right panel). For parallel bundles of treadmilling filaments, image subtraction results in fluorescent spots corresponding to FtsZ binding or detaching from a filament bundle allowing to visualize polymerization (growth, green) and depolymerization (shrinkage, magenta) trajectories. We expect that spots corresponding to growing and shrinking ends have similar intensities and closely follow each other. In contrast, for antiparallel bundles of treadmilling filaments, the spots corresponding to growth and shrinkage would move in opposite directions and cross each other. **b** Raw image of FtsZ filament network and the corresponding differential image overlaid showing growing (green) and shrinking (magenta) ends without ZapA and (**c**) in the presence of 6.0 μM ZapA. Orange arrows indicate treadmilling direction. Right: Intensity distribution of fluorescence spots generated from a differential time-lapse movie of FtsZ/FtsA filaments and with 6.0 μM ZapA (**c**). Insets depict the mean normalized intensities and standard deviation (shaded error bands) of spots versus trajectory length. The presence of ZapA does not change fluorescent spot intensity and accordingly filament orientation. Scale bars: large image, 5 μm; small image, 2 μm. Source data available at Figshare [10.6084/m9.figshare.10347062.v1].

To obtain information about the relative orientation of FtsZ filaments within the bundles, we needed to develop a method to measure the polarity of filaments within dynamic bundles. To this end, we first constructed differential time-lapse movies, where we computed the intensity differences between consecutive frames after applying a spatiotemporal low-pass filter similar as in previous studies on microtubule dynamics^20,21^ (Fig. 4a, b; Supplementary Movie 2; methods for details). This image-processing step removes static objects from the movie while regions where the image changes give rise to either a positive or negative signal, corresponding to areas where the intensity has either increased or decreased between the subsequent frames. Indeed, with only FtsZ and FtsA present in the system, this computation step produced fluorescent spots corresponding to growing and shrinking ends of filament bundles while moving unidirectionally along bundles (Supplementary Movie 2). Using particle tracking software to automatically detect and analyze these spots, we also found that they had roughly constant intensity along their trajectories (Fig. 4b). This observation of directionally moving spots of constant intensity is consistent with a predominant polar orientation of filaments within a treadmilling bundle (Fig. 4a, left panel). Next, we performed this analysis for filament bundles formed in the presence of ZapA. In case of a rearrangement of the filaments into anti-parallel bundles we expected these fluorescent spots to have different intensities and behavior (Fig. 4a, right panel). However, these fluorescent spots showed only a slightly reduced intensity and similar intensity traces along their treadmilling trajectories with and without 6.0 μM ZapA (Supplementary Movie 3, Fig. 4c). Together, these findings show that filaments maintain their parallel, polar orientation even in the presence of ZapA.

### ZapA does not affect FtsZ treadmilling velocity

Next, we investigated whether the presence of ZapA slows down the treadmilling velocity of FtsZ filaments. Previous in vitro studies reported that ZapA inhibits the GTPase activity of FtsZ, suggesting that it impairs treadmilling^15–17^. However, a decrease in filament turnover could perturb the spatiotemporal dynamics of cell wall synthases in vivo, as their motion is driven by FtsZ treadmilling. Knocking out individual Zap proteins in vivo did not show any significant change in the FtsZ treadmilling velocity^5^, but due to their overlapping functions it is difficult to rule out the possibility of other FtsZ-associated proteins compensating for the loss of crosslinking in these mutants. Accordingly, the direct influence of FtsZ-binding proteins on filament dynamics is unknown.

**Fig. 5:**
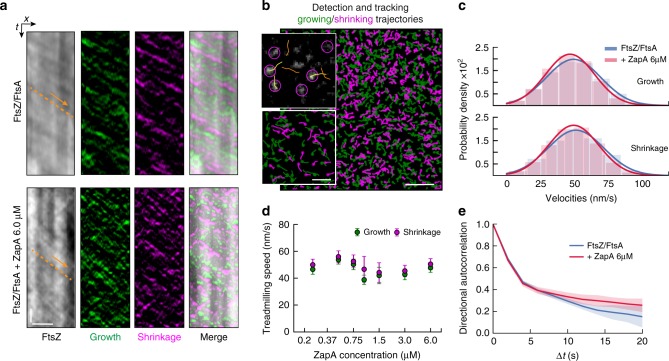
ZapA does not change treadmilling speed of FtsZ filaments. **a** Kymographs obtained along the orange line in Fig. 4b. Images represent the channel with the raw data, the two differential channels (growth and shrinkage trajectories) and the merged image. Arrows indicate treadmilling direction. Scale bars, *t* = 20 s, *x* = 3 μm. **b** Snapshot showing an example of detection (purple circles) and tracking (yellow/orange lines) of fluorescent spots and the respective generated growing and shrinking trajectories, respectively. First position of each trajectory is indicated with circles. Scale bars: large image, 5 μm; small image, 2 μm. **c** Velocity distribution of filament growth (upper panel, FtsZ/FtsA, ν_+_ = 50.5 ± 20.1 nm/s, FtsZ/FtsA + ZapA, ν_+_ = 47.8 ± 18.2 nm/s, *n* = 10) and shrinkage (lower panel, FtsZ/FtsA, ν_−_ = 53.0 ± 20.7 nm/s, FtsZ/FtsA + ZapA, ν_−_ = 50.4 ± 18.6 nm/s, *n* = 10). Both show a normal distribution without any significant difference in the mean. **d** Increasing concentrations of ZapA had no effect on the mean velocity of FtsZ filaments at either end (*p* > 0.05; Supplementary Table 1). **e** Directional autocorrelation revealed that directionality was increased in the presence of 6 μM ZapA (red) when compared to FtsZ/FtsA (blue). Data shows the average autocorrelation curve from individual experiments (shaded bars = std, *n* = 10). Source data available at Figshare [10.6084/m9.figshare.10347062.v1].

To investigate how ZapA affects FtsZ dynamics we obtained kymographs along bundles of treadmilling filaments from our raw data as well as from differential time-lapse movies to visualize growing and shrinking ends (Fig. 5a). Here, the treadmilling velocity corresponds to the slope of diagonal lines seen in the kymograph (e.g orange arrow). Interestingly, we did not find an obvious difference in velocities for filaments with or without ZapA. To precisely quantify treadmilling velocities of FtsZ filaments in an unbiased manner, we analyzed the treadmilling trajectories obtained from the automated detection of fluorescent spots and single particle tracking (Fig. 5b; methods for details). This method allowed us to calculate the growth (***ν***_+_) and shrinkage velocities (***ν***_−_) of thousands of treadmilling tracks simultaneously (Fig. 5c, d and Supplementary Fig. 3a). In the absence of ZapA, we found ***ν***_+_ and ***ν***_−_ to be normally distributed with similar mean values (***ν***_+_ = 50.5 ± 3.0 nm/s and  ***ν***_−_ = 53.0 ± 3.6 nm/s, *n*  = 10), consistent with a treadmilling behavior (Fig. 5c, blue). Importantly, we found similar values in the presence of 6.0 μM ZapA (***ν***_+_ = 47.8 ± 3.45 nm/s and ***ν***_−_ = 50.4 ± 4.1 nm/s, *n * = 10) (Fig. 5c, red), for all other ZapA concentrations (Fig. 5d) and for both ZapA mutants tested in our experiments (Supplementary Fig. 3c). Accordingly, we found that ZapA has no effect on the treadmilling velocity of membrane-bound FtsZ filaments.

In addition, we performed a directional autocorrelation analysis on FtsZ treadmilling trajectories, which provides information about the directional persistence of the particles within each track (methods for details). In agreement with directional motion, the correlations for treadmilling trajectories were always positive and followed a slow decay (Fig. 5e). Interestingly, we observed that the directionality was preserved for longer time delays in the presence of ZapA indicating straighter treadmilling trajectories. This observation is also consistent with the observed decrease in curvature and increase in spatial order (Fig. 2).

### ZapA does not affect filament length

Next, we wondered if the increase in spatial order and decrease in curvature with ZapA was due to an increased mean length of FtsZ filaments. Directly measuring the length of individual filaments within dense bundles is not possible by light microscopy, but as the spots for growth and shrinkage of a given bundle correspond to the two different ends of filaments, we reasoned that the distance between them provides an estimate of the mean bundle length. Using this approach, we estimated the bundle length to be around 500 nm independent of the presence of ZapA (Supplementary Fig. 3b).

**Fig. 6:**
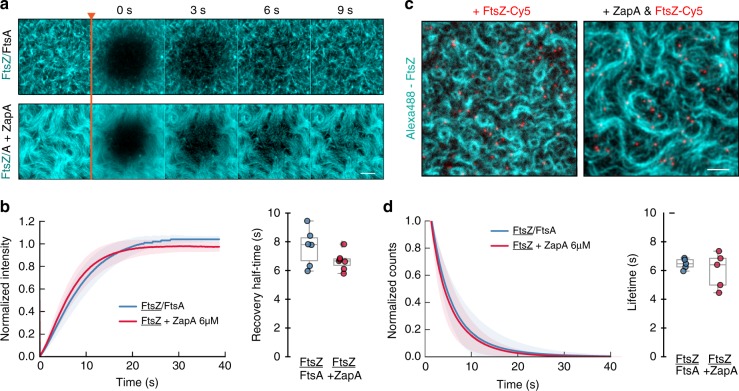
ZapA does not change length of FtsZ filaments. **a** Snapshots showing fluorescence recover after photobleaching (FRAP) experiments for FtsZ filaments without (upper panel) and in the presence (lower panel) of 6 μM ZapA. **b** Half-time recovery of FtsZ was not affected by the addition of ZapA (7.63 ± 1.30 and 6.66 ± 0.64 s, respectively). **c** Snapshot from single molecule tracking experiments of FtsZ (red) with and without 6 μM ZapA. Scale bars, 5 μm **d** Lifetime distribution of FtsZ does not change significantly with ZapA (FtsZ = 6.59 ± 0.28 s, blue, *n* = 5; FtsZ with ZapA = 6.01 ± 1.24 s, red, *n* = 6). Source data available at Figshare [10.6084/m9.figshare.10347062.v1].

The length of treadmilling filaments can also be estimated by calculating the product of the treadmilling velocity and the lifetime of FtsZ monomers within filaments. Accordingly, as the treadmilling velocity was unchanged, an increase in filament length should go along with a significant decrease in filament turnover. We analyzed the turnover rate of FtsZ in fluorescence recovery after photobleaching (FRAP) experiments (Fig. 6a; Supplementary Movie 4) and found a mean recovery half-time of 7.63 ± 1.30 s (blue, *n* = 6) in the absence of ZapA and 6.66 ± 0.64 s (red, *n* = 7) with 6.0 μM ZapA, suggesting that ZapA does not change the filament length (Fig. 6b; two-tailed t test with *p* = 0.47).

To further corroborate these results, we performed single molecule experiments, where we added small amounts of a Cy5-labelled FtsZ to a background of Alexa488-labelled FtsZ (Fig. 6c; Supplementary Movie 5). This allowed us to directly measure the lifetime of FtsZ monomers in the treadmilling filaments. In agreement with our FRAP experiments, we found no difference in the lifetimes of FtsZ with and without ZapA (FtsZ, 6.59 ± 0.28 s, *n* = 5; FtsZ + ZapA, *n* = 6.01 ± 1.24 s, *n* = 6; two-tailed *t* test with *p* = 0.14) (Fig. 6d, blue and red, respectively).

Together with our quantification of the treadmilling velocity, our findings suggest a mean FtsZ filament length between 341.6 ± 50.0 and 412.0 ± 37.7 nm, when only FtsZ and FtsA were present, and between 302.4 ± 72.9 and 351.8 ± 76.2 nm with 6.0 μM ZapA, again showing that ZapA does not change the filament length. These values are similar to the estimated mean length obtained from the distance between growing and shrinkage spots (Supplementary Fig. 3b) and about three times longer than what has been suggested for filaments in vivo^22^.

Together, these experiments and analyses show that despite its strong effect on the architecture and reorganization dynamics of the FtsZ filament network, ZapA does not slow down or enhance the underlying polymerization dynamics (Supplementary Table 3). Furthermore, the presence of ZapA does not affect the relative orientation of FtsZ filaments towards each other or their length, while it increases the directional persistence of treadmilling.

### ZapA binds transiently to FtsZ filaments

**Fig. 7:**
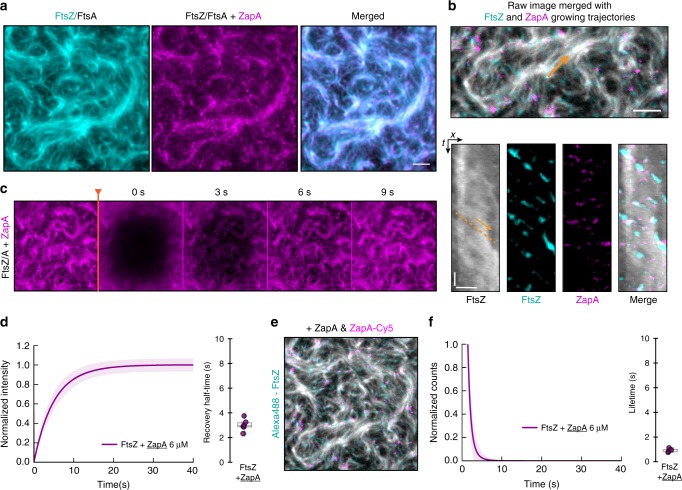
ZapA binds only transiently to FtsZ filaments. **a** Dual-color TIRF micrograph showing co-localization of Alexa488-FtsZ (cyan) and ZapA-Cy5 (magenta). **b** Raw image of FtsZ filament network overlaid with growing trajectories of FtsZ (cyan) and ZapA (magenta) from differential imaging (scale bar 5 μm). Kymographs are obtained along the orange arrow, which indicates treadmilling direction. Scale bars, *t* = 20 s, x = 3 μm. **c** Snapshots showing FRAP experiments of Cy5-labelled ZapA pattern. **d** Half-time recovery of Cy5-labelled ZapA showed a much faster recovery (ZapA = 3.01 ± 0.47, *n* = 6) when compared with FtsZ (Fig. 6b). **e** In contrast with FtsZ (Fig. 4d), the lifetime of ZapA was much shorter (ZapA = 0.95 ± 0.14, *n* = 5). Source data available at Figshare [10.6084/m9.figshare.10347062.v1].

Next, we sought to better understand how ZapA could change the architecture of FtsZ filaments without slowing down their treadmilling dynamics. To this end, we prepared a fully functional C-terminally labeled version of ZapA (Supplementary Table 4), ZapA-Cy5, which allowed us to image its dynamic behavior simultaneously with FtsZ. We found that, as in vivo, ZapA co-localized with FtsZ bundles on the membrane (Fig. 7a; Supplementary Movie 6). Having the capability to image at higher spatiotemporal resolution than in the living cell, we also observed that ZapA showed a more discontinuous appearance (Fig. 7a; Supplementary Movie 6) than FtsZ. Still, our dual-color time lapse movies, corresponding differential images (Supplementary Movie 7) and kymographs (Fig. 7b) revealed that ZapA was able to follow the treadmilling dynamics of FtsZ filaments even though the corresponding trajectories were much shorter. These results suggest that even though ZapA co-localized with treadmilling FtsZ filaments, ZapA alone does not function as a direct readout for treadmilling dynamics.

We then analyzed the turnover rate of ZapA in FRAP (Fig. 7c; Supplementary Movie 8) and single molecule experiments (Fig. 7e, Supplementary Movie 9) and found a half-recovery rate of 3.01 ± 0.47 s (Fig. 7d, *n* = 6) and a single-molecule lifetime of 0.94 ± 0.14 s (Fig. 7f; *n* = 5). Accordingly, ZapA tetramers are incorporated into membrane-bound FtsZ bundles about 2 to 8 times shorter than FtsZ monomers despite having multiple binding sites for FtsZ.

Together, these results show that the strong influence on the large-scale organization of the FtsZ filament network is the result of cooperative, transient binding events between multivalent ZapA tetramers and FtsZ filaments. Furthermore, we think that this fast turnover of components of the Z-ring can help this cytoskeletal structure to continuously adapt to the shrinking diameter of the cell during constriction.

## Discussion

FtsZ has long been known as the main organizer of bacterial cell division by playing two important roles: First, as FtsZ polymerization into the Z-ring initiates the assembly of the division machinery, its intracellular position defines the location of cell division. Second, FtsZ treadmilling was found to drive a circumferential motion of peptidoglycan synthases, which is required for the homogeneous distribution of cell wall synthesis at the division site. In this study, we were interested in understanding how the components of the cell division machinery are able to contribute to these two roles. Specifically, we wanted to know how the FtsZ-associated protein ZapA can contribute to the stability of the Z-ring and precision of cell division, while still allowing FtsZ filaments to continuously turn over.

**Fig. 8:**
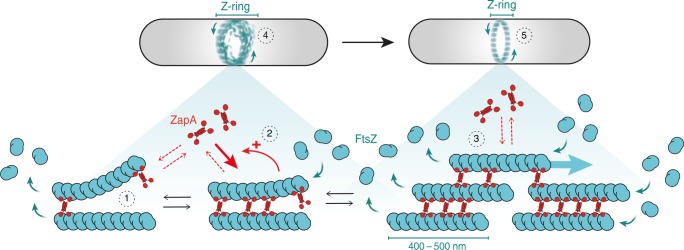
A model for the influence of ZapA and its role for Z-ring maturation. Binding of ZapA aligns treadmilling FtsZ filaments (1), which in turn facilitates its binding, giving rise to cooperativity (2). At saturation, four molecules of FtsZ bind to one tetramer of ZapA (1:1 monomer ratio, [FtsZ] = [ZapA]) in parallel increasing the bundle width, persistence and alignment of FtsZ filaments (3). Treadmilling is not affected due to the transient binding of ZapA. Together, ZapA leads to a shift from a network of less ordered and highly dynamic FtsZ filaments (4) into a more well-defined track, acting as an additional fine-tune mechanism for a proper Z-ring alignment (5) without compromising treadmilling dynamics.

Using an in vitro reconstitution approach that allowed us to study the dynamic behavior of membrane-bound FtsZ filaments bundles directly, we found that ZapA aligns FtsZ filaments in parallel, which gives rise to a straightening and stabilization of filament bundles and as a consequence increases the spatial order and spatiotemporal persistence of the large-scale filament network. Importantly, this transition from a dynamic state of low spatial order to a persistent state of high order happens within a narrow range of ZapA concentrations. What could be an explanation for this cooperative switch? Due to its central four-helix bundle, the ZapA tetramer can be assumed to have a highly rigid structure. We therefore expect that only in the case of straight, parallel filaments all four FtsZ-binding domains of a ZapA tetramer would be in contact with an FtsZ monomer. Accordingly, binding of ZapA aligns and straightens FtsZ filaments, which in turn can increase their affinity to ZapA tetramers. As a result, ZapA stimulates its own recruitment (Fig. 8). We believe that this ultrasensitive response plays an important role in the maturation of the cell division machinery as it ensures the correct alignment of FtsZ filaments following Z-ring assembly and prior to cell wall remodeling. As ZapA was found to increase the stiffness of FtsZ gels formed in vitro^7^, the switch that we observe here could also have consequences for the mechanical properties of the Z-ring.

Our experiments show that FtsZ, FtsA, and ZapA self-organize into highly ordered cytoskeletal patterns on the membrane. How does this system compare to filament networks of other cytoskeletal proteins? Similar to gels of actin filaments and molecular motors, the structures described here exist out of equilibrium. In contrast to these active gels, however, the FtsZ filament network is driven out of equilibrium due to the continuous consumption of energy during treadmilling and not by the activity of motor proteins. This creates the important distinction that the components of an FtsZ active gel do not necessarily need to move relative to each other in order to rearrange the network. As for ZapA, bundling by actin crosslinkers is known to modulate the architecture and mechanical properties of actin filament networks even if they interact transiently^23^. Like ZapA, actin crosslinkers were also found to bind cooperatively to actin filaments^24,25^. However, while actin crosslinkers were found to slow down actin depolymerization^26^, FtsZ treadmilling rates stay constant even at saturating concentrations of ZapA. Accordingly, the switch in FtsZ filament organization that we observed here can also be interpreted as a phase transition that originates from the cumulative effect of multiple weak interactions between multivalent proteins that continuously turn over^27^. The fact that ZapA binds only transiently to FtsZ filaments is also an important distinction to microtubule crosslinkers such as Ase1, whose activity depends on persistent binding and diffusion on two antiparallel microtubule lattices. Importantly, this property allows Ase1 to generate entropic forces by maximizing the microtubule overlap and the accessible area for diffusible crosslinker^28,29^. Therefore, we believe that the cytoskeletal networks of FtsZ, FtsA and ZapA represent a novel and distinct form of active biological matter despite the apparent absence of mechanical stresses that are usually present in gels composed of filaments and molecular motors^30–32^.

To conclude, our observations show that ZapA is able to increase the precision and robustness of the Z-ring, without sacrificing the performance of the cell division machinery.

## Methods

### Reagents and protein biochemistry

All chemicals and protein biochemistry protocols can be found in the Supplementary Information.

### Self-organization assay

To prepare small unilamellar vesicles (SUVs), DOPC and DOPG dissolved in chloroform were transferred into a glass vial to a final concentration of 5 mM (70:30 molarity ratio). Using a stream of nitrogen, the mixture was dried to form a thin film of lipids and transferred to a vacuum desiccator for 3 h to completely remove any residual chloroform. The lipids were then rehydrated in swelling buffer (50 mM Tris-HCl pH 7.5, 300 mM KCl) and incubated at 37 C for 30 min. The lipid film was then resuspended by vortexing vigorously to obtain multilamellar vesicles of different sizes and frozen using liquid nitrogen. To obtain small unilamellar vesicles (SUVs), the suspension was subjected to 5–10 freeze-thaw cycles followed by tip sonication. Vesicles were kept at −20 °C in aliquots of 30 μl up to two months.

To prepare the reaction chamber, glass coverslips were first cleaned by sonicating (>15 min) in 2% Hellmanex II solution (Hellma), followed by extensive washing and sonication in milliQ H_2_O and storage in 95% reagent-grade ethanol for up to one week. Before use, glass coverslips were blown dry with compressed air and cleaned in an air plasma for 10 min. The reaction chamber was prepared by attaching a plastic ring (top of Eppendorf tube) on a cleaned glass coverslip using ultraviolet glue (Norland optical adhesive 88).

To form supported lipid bilayers (SLBs), a SUVs suspension was diluted to 1 mM in swelling buffer and CaCl_2_ was added to a final concentration of 2 mM. From this mix, 50 μl were added to each reaction chamber. Vesicles adsorb to the surface, rupture and fuse with the clean hydrophilic glass to form a flat bilayer, assisted by the presence of CaCl_2_. After 1 h of incubation at RT, 50 μl of swelling buffer was added to each chamber (final volume = 100 μl) and sample was rinsed several times with 200 μL of reaction buffer (50 mM Tris-HCl pH 7.5, 150 mM KCl, 5 mM MgCl_2_).

To performed the self-organization assay, all experiments were initiated by first adding an oxygen scavenger system to the reaction chamber (0.2% d-Glucose, 0.016 mg/ml glucose oxidase, 0.002 mg/ml catalase, 1 mM dithiothreitol and 0.25 mg/ml trolox) to prevent photobleaching. Then, FtsZ (with 30% Cy5- or Alexa488-labeled protein) and FtsA were added to the reaction chamber in a final concentration of 1.5 and 0.5 μM, respectively. To trigger polymerization and membrane recruitment of FtsZ, ATP and GTP were added to the system to a final concentration of 4 mM. ZapA was added in different concentration ranges before the addition of GTP.

### Fluorescence microscopy

All experiments were performed on an Inverted multipoint total internal reflection fluorescence (TIRF) microscope (TILL Photonics) equipped with dual camera TIRF objectives (Andor 897straight (X-8449) 512 × 512 pixel and Andor 897 (X-8533) 512 × 512 pixel) and an image splitter (Andor Tucam) equipped with a long pass of 580 and 640 nm. Alexa488 and Cy5 dyes were excited using 488 nm and 642 nm laser lines, respectively, and the emitted light was filtered by an Andromeda quad-band bandpass filter. Images were typically obtained every 2 s, with 50 ms exposure to minimize photobleaching and using a ×100 Olympus TIRF (NA = 1.49 DIC) objective.

### Image processing

For image processing, image stacks were imported using FIJI software^33^. Prior to image analysis and visualization (supplemental movies*)* acquired time-lapse movies were normalized to create a constant overall intensity and compensate for the increasing intensity over time due to protein binding to the membrane.

### Quantification of bundle width

To estimate the width of membrane-bound FtsZ bundles, fluorescence images were binarized using the adaptive threshold plugin for ImageJ. This plugin corrects for non-homogeneous background intensities, overcoming the limitation of conventional threshold methods. The background was removed using a local threshold corresponding to the mean intensity of an area of 20 × 20 pixels without any further subtraction. The binarized time lapse movie was then processed with the despeckle filter in ImageJ to remove small particles. Next, we calculated the Euclidean Distance Map (EDM) of every frame using the Distance Mapping function of ImageJ. This transformation results in a grey scale image, where the grey value of each pixel represents the shortest distance to the nearest pixel in the background. Accordingly, bundle widths correspond to the local peak intensities multiplied by 2. The mean bundle width for every frame of the movie was calculated by identifying the peak intensities for each line and column of the image using a MATLAB script. This value could then be plotted as a function of time. The characteristic bundle width for different concentrations of ZapA and respective mutants was obtained by taking the time-average at steady state.

### Architecture analysis

For a quantitative description of the filament architecture, we first calculated the orientation field of the pattern for each frame of a time-lapse movie using a gradient squared tensor approach. From these time-dependent orientations we calculated two independent parameters: curvature, *k* given by the rate of change in the local orientation at each pixel position, and the spatial order, *S*(*r*), given by the spatial correlation of the angles as a function of an increasing distance *r*. To describe time-dependent curvature changes, the distribution of curvature values from each frame were fitted to a single exponential and the mean curvature for each image *k* was given by the mean characteristic length scale of the exponential. Likewise, temporal changes in the spatial order of the system were characterized by the distance *r* at which *S*(*r*) = 0.5 (intersection) at a given time. A more detailed description of the theoretical background is provided in the Supplementary Information.

### Image autocorrelation analysis

To quantify the reorganization dynamics of the membrane-bound FtsZ filament network, we performed a temporal correlation analysis using the ImageCorrelationJ plugin for ImageJ^34^. This method calculates the Pearson cross-correlation coefficient (PCC) between two frames with an increasing time lag between them (∆t)^34^. The mean intensities in local areas of 3 × 3 pixels were used to calculate the PCC between two different images, which was then plotted as a function of ∆t (Fig. 3b). For highly dynamic image pattern, the PCC typically decays rapidly with ∆t, but slowly for persistent structures. The corresponding correlation curve was fitted to a mono-exponential decay, and the half-time (correlation time, *τ*) was used to compare a set of individual experiments.

### Treadmilling velocity analysis

To quantify treadmilling dynamics, we have developed an automated image analysis based on differential image stacks and automated particle tracking using TrackMate^35^, a particle tracking toolbox available for ImageJ. This methodology works by first subtracting the intensity of two consecutive frames from one another to create a new time-lapse movie containing directionally moving fluorescent spots, either corresponding to growth or shrinkage of filaments at a given position. Next, using TrackMate we identify and track these spots to reconstruct their trajectories and obtain velocity and directionality using a custom python script. A more detailed description of the protocol, as well as a supporting code can be found in Supplementary Information.

### Single molecule tracking

Single molecule experiments were performed as described in ref.^36^. Briefly, individual FtsZ monomers were resolved at single molecule level by mixing small amounts of Cy5-labelled FtsZ (<2 nM) to a background of 1.5 μM Alexa488-labelled FtsZ. To track individual ZapA molecules, we followed a similar strategy by adding small amounts of ZapA-Cy5 to a background of unlabeled ZapA, always in the presence of Alexa488-FtsZ 1.5 μM. Experiments were typically recorded using minimal laser power to visualize single molecules, with an exposure time of 50 ms and a varying acquisition time (114 ms to 1 s) for further photobleaching correction^36^. Single molecules were identified and tracked using the automated particle-tracking platform TrackMate^35^, with the following parameters: a particle size of 0.5 μm, particles localized for at least two frames were considered, linking max distance and gap closing distance of 0.5 μm, and two frames for the gap closing max. The mean lifetime of single molecules was obtained by fitting a mono-exponential decay to the lifetime distribution.

### Fluorescence recover after photo bleaching

For Fluorescence recover after photo bleaching (FRAP) experiments, we allowed the system to reach the steady state (15 min) and a small area of the membrane was bleached using a high laser intensity. To obtain the half-time of the recovery, a Jython macro script for ImageJ (Image Processing School 8 Pilsen 2009) was used to fit the fluorescence recovery to *I*(*t*) = *a*(1 − exp(−*bt*))), where I(t) is the intensity value corrected for photobleached effects. FRAP experiments were acquired with an exposure time of 50msec and an acquisition time of one frame every 114 msec.

### Statistical analysis

For statistical analyses of box plots data, a two-tailed Student’s *t*-test was performed using a python script based on scipy library. A *p*-value of <0.05 was considered as statistically significant. Boxes are represented from 25th to 75th percentiles, the whiskers extend 1.5x the standard deviation and the mean value is plotted as a line in the middle of the box. Each titration curve was fitted to a Hill equation using a custom written Python script. Confidence intervals for the Hill coefficient and EC50 were estimated by bootstrapping the non-linear regression in 1000 iterations^37^.

### Reporting summary

Further information on research design is available in the Nature Research Reporting Summary linked to this article.

## Supplementary information

Supplementary Information

Peer Review

Reporting Summary

Description of Additional Supplementary Files

Supplementary Movie 1

Supplementary Movie 2

Supplementary Movie 3

Supplementary Movie 4

Supplementary Movie 5

Supplementary Movie 6

Supplementary Movie 7

Supplementary Movie 8

Supplementary Movie 9

## Data Availability

Source data for all figures presented in the paper have been provided in a data source table available at Figshare at 10.6084/m9.figshare.10347062.v1. Uncompressed raw movie files are available at Figshare at 10.6084/m9.figshare.10333079.v2. All other data supporting the findings of this study are available from the corresponding author upon request.

## Code availability

To quantify treadmilling dynamics, we developed an automated image analysis protocol based on ImageJ and custom Python scripts. All code source files are available online: https://github.com/paulocaldas/Treadmilling-Speed-Analysis or upon request from the corresponding author.
