# Supplementary material for: Cooperative ordering of treadmilling filaments in cytoskeletal networks of FtsZ and its crosslinker ZapA
Source: Nat Commun. 2019 Dec 17;10:5744. doi: 10.1038/s41467-019-13702-4 (PMC6917738; doi:10.1038/s41467-019-13702-4)
Supplement: Supplementary file 1 — Supplementary Information [file 41467_2019_13702_MOESM1_ESM.pdf]

Supplementary Information

Cooperative ordering of treadmilling filaments in cytoskeletal networks of FtsZ and its crosslinker ZapA

Authors

Paulo Caldas[1], Mar Lopez-Pelegrin[1], Daniel J.G. Pearce[2],
Nazmi B. Budanur[1], Jan Brugués[3,4,5,6] and Martin Loose[1]

[1] Institute of Science and Technology Austria, Klosterneuburg, Austria;
[2] School of Chemistry and Biochemistry, University of Geneva, Switzerland;
[3] Max Planck Institute of Molecular Cell Biology and Genetics, Dresden, Germany;
[4] Max Planck Institute for the Physics of Complex Systems, 01187 Dresden, Germany,
[5] Centre for Systems Biology Dresden, 01307 Dresden, Germany
[6] Cluster of Excellence Physics of Life, TU Dresden, 01062 Dresden, Germany

Corresponding author

Martin Loose.
Email: martin.loose@ist.ac.at; Telephone: +43 (0) 2243 9000 6301.

Supplementary Methods

Reagents and Chemicals

Phospholipids used in this paper, DOPC (1,2-dioleoyl-sn-glycero-3-phosphocholine) and DOPG (1,2-dioleoyl-snglycero-3-phospho-(1'-racglycerol)), were purchased from Avanti Polar Lipids (Alabaster, AL) and kept at -20°C as 25 mg/ml stock solutions in chloroform; Sulfo-Cyanine-5-maleimide (Cy5) was acquired from Lumiprobe and Alexa Fluor® 488 C5-Maleimide (Alexa488) was acquired from ThermoFisher Scientific; Nucleotides were acquired from ThermoFisher Scientific or Jena Bioscience; Precision cover glasses for the homebuilt chambers were obtained from VWR (thickness No. 1.5H, 24 x 50); *E.coli* strains were obtained from Lucigen; Strep-Tactin resin was acquired from Iba lifesciences and Nickel resins were purchased from ThermoFisher Scientific (HisPur™ Ni-NTA resin) or Macherey-Nagel (Protino Ni-TED resin); All the remaining reactants and salts were obtained from Sigma, Merck, or Invitrogen and were of analytic or spectroscopic grade.

Protein Biochemistry

Purification and labelling of FtsZ

FtsZ (UP P0A9A6) was cloned into a pTB146-derived vector which attached a N-terminal His_6-SUMO tag plus seven additional amino acids (AEGCGEL) that provide a cysteine residue for further fluorescent labelling (pML45, His_6-SUMO-GCG-FtsZ). *E.coli* C41 (DE3) cells were transformed with pML45 and grown in TB medium supplemented with ampicillin at 37°C, until cells reached an OD600 of 0.8. After the addition of IPTG to a final concentration of 1mM, cells grew for 5h at 37°C. Cells were harvested by centrifugation, pellets were frozen in liquid nitrogen and kept at -80°C until further use.

For purification, pellets were thawed and resuspended in FtsZ buffer (50 mM Tris-HCl pH 8.0, 500mM KCl, 2mM β-mercaptoethanol, 10% glycerol) supplemented with 20mM imidazole and cOmplete EDTA-free protease inhibitors cocktail (1 tablet/50ml, Roche)) followed by incubation at 4°C for 15 min. Cells were lysed using a cell disruptor (Constant Systems) at a pressure 1.36 kbar and incubated with 1 mg/ml DNase I (Sigma-Aldrich) and 2.5 mM $MgCl_2$ for 15min. The lysate was then centrifuged (30 min, 60,000 g, 4°C) and the supernatant was incubated with nickel agarose resin (HisPur Ni-NTA resin, Thermo Scientific) for 60 min at 4°C. The resin was extensively washed with FtsZ buffer supplemented with 20 mM imidazole and 30 mM imidazole. The fusion protein was eluted with FtsZ buffer supplemented with 250 mM imidazole. Fractions were evaluated by SDS-PAGE (stained with Coomassie Blue) and peak fractions containing His_6-SUMO-GCG-FtsZ were pooled and incubated with His_6-tagged SUMO protease (His_6-Ulp1) during an overnight dialysis into FtsZ cleavage buffer (50mM Tris-HCl pH 8.0, 300 mM KCl and 10% glycerol). The digested sample was passed several times

through Ni-NTA resin, to remove His_6 containing molecules. The flow through was collected and active protein was enriched by sedimentation of FtsZ filaments. For this, the protein was dialyzed into FtsZ polymerization buffer (50 mM PIPES pH 6.7, 10 mM $MgCl_2$) and incubated with $CaCl_2$ and GTP for 15 min at 30°C. The solution was then centrifuged (2 min, 20,000xg, RT) and a clear gel-like pellet containing polymeric FtsZ was obtained. The pellet was resuspended into FtsZ storage buffer (50mM Tris-HCl pH 7.4, 50 mM KCl, 1mM EDTA and 10% glycerol) and incubated with 100×molar excess of 7 Tris(2-carboxyethyl)phosphine hydrochloride (TCEP) for 20 min at RT for cysteine-labelling. Five times molar excess of a thiol-reactive dye (Alexa488 or Cy5–maleimide) was added to the solution and incubated overnight at 4°C during dialysis into FtsZ storage buffer. Finally, labelled FtsZ was loaded on a PD10 desalting column to remove $CaCl_2$, GTP and free dye. Purified FtsZ was aliquoted, flash frozen in liquid nitrogen and kept at -80°C until usage.

Purification of FtsA

The gene coding for FtsA (UP P0ABH0) was cloned into a modified pTB146 vector. The resulting vector, pML60, encodes for FtsA with an N-terminal His_6-SUMO-pentaglycine tag. *E.coli* C41 (DE3) cells were transformed with pML60 and grown in 2xYT medium supplemented with ampicillin, at 37°C, until they reached an OD600 of 0.6-0.8. After the addition of IPTG to a final concentration of 1mM, cells grew overnight at 18°C. Cells were harvested by centrifugation, pellets were frozen in liquid nitrogen and kept at -80°C until further use.

For purification, cells were thawed and resuspended in FtsA buffer (50 mM Tris-HCl pH 8.0, 500mM KCl, 10 mM $MgCl_2$) supplemented with 1 mg/ml lysozyme, cOmplete EDTA-free protease inhibitors cocktail (1 tablet/ 50 ml, Roche), 1 mg/ml DNase (Sigma-Aldrich) and 0.5mM DTT. Cells were then lysed using a cell disruptor (Constant Systems) at a pressure of 1.36kbar, centrifuged (60,000xg, 45min at 4°C) and the supernatant was incubated with Nickel agarose beads (Protino Ni-TED, Macherey-Nagel) for 1h at 4°C.

The resin was extensively washed with FtsA buffer and FtsA buffer supplemented with 5mM imidazole. The fusion protein was then eluted with FtsA buffer supplemented with 250mM imidazole and fractions were evaluated by SDS-PAGE (stained with Coomassie Blue). Peak fractions containing His-SUMO-GGGGG-FtsA were pooled and the buffer was exchanged into FtsA storage buffer (50 mM Tris-HCl pH 8.0, 500 mM KCl, 10 mM $MgCl_2$, 0.5 mM DTT, 20% glycerol). The His_6-SUMO tag was cleaved by incubating His-SUMO-GGGGG-FtsA with His_6-Ulp1 for 90min at 30°C. The sample was then passed several times through Protino Ni-TED resin, previously equilibrated with FtsA storage buffer, to remove His_6-containing molecules. Protein was aliquoted, flash frozen in liquid nitrogen and kept at -80°C until usage.

Purification of ZapA and Mutants

ZapA (UP P0ADS2) was cloned into a modified pTB146 vector attached to an N-terminal Twinstrep-SUMO tag (pML130, Twinstrep-SUMO-ZapA). *E.coli* BL21 (DE3) cells were transformed with pML130 and grown in TB medium supplemented with ampicillin, at 37°C, until cells reached an OD600 of 0.6-0.8. After the addition of IPTG to a final concentration of 1 mM, cells grew for 4h at 37°C. Cultures were harvested, and pellets were frozen in liquid nitrogen and kept at -80°C until further use.

For purification, cells were thawed and incubated with ZapA buffer (50 mM HEPES pH 7.5, 300mM KCl, 10% glycerol) supplemented with 2mM β-mercaptoethanol and cOmplete EDTA-free protease inhibitor tablets (1 tablet/50 ml, Roche Diagnostics) for 15 min at 4°C. Cells were then lysed using a cell disruptor and incubated in the presence of 1 mg/ml of DNase (Sigma-Aldrich) and 2.5 mM $MgCl_2$ for 15min. The lysate was centrifuged (30 min, 60,000 g, 4°C) and the supernatant was incubated with strep-tactin resin (Strep-Tactin® resin, Iba Lifesciences) for 30min at 4°C. Beads were extensively washed with ZapA buffer and the fusion protein was eluted using ZapA buffer supplemented with 50mM biotin. Peak fractions of Twinstrep-SUMO-ZapA were identified by SDS-PAGE (stained with Coomassie Blue) and pooled. The Twinstrep-SUMO tag was cleaved with His_6-Ulp1 during an overnight dialysis into ZapA storage buffer (50mM Tris-HCl pH 7.5, 50 mM KCl, 10% Glycerol). The solution was then passed through Ni-NTA and strep-tactin resins to remove His_6-Ulp1 and free twinstrep-SUMO tag, respectively. ZapA was aliquoted, flash frozen in liquid nitrogen and kept at -80°C until usage.

For labeling purposes, ZapA was additionally cloned into a vector which attached 5 residues (LPETG) to the C-terminus of the protein, pMAR11b (TwinStrepSUMO-ZapA-LPETG). ZapA-LPETG was expressed and purified as the wildtype protein followed by Sortase-mediated labelling (1). Specifically, ZapA-LPETG was incubated with 0.5 mM of labelled peptide GGGC-Cy5 and 10µM Sortase 7M (purified using pET30b-7M SrtA, a gift from Hidde Ploegh, Addgene plasmid #51141) in a final concentration of 50 µM. The reaction was carried during an overnight dialysis into ZapA storage buffer at 4°C. Sample was further purified from free labelled peptide and Sortase by size-exclusion chromatography using a Hi/Load 16/600 Superdex 75 column (GE Healthcare) previously equilibrated with ZapA storage buffer. Labelled ZapA was collected, aliquoted and flash frozen in liquid nitrogen. ZapA mutants, R46A and I83E, were obtained by site-directed mutagenesis (pMAR9 and pMAR10b, respectively) and purified following the same procedure as the wild type protein.

Architecture analysis

For a quantitative description of the filament architecture, we first calculated an orientation field of the pattern by calculating a gradient squared tensor at every position of a fluorescence micrograph(2, 3). This analysis was performed either using the OrientationJ plugin for ImageJ or using a custom python code based on the *scikit-image* package (Fig. 2A). For this method, a unit vector $u_\theta = (cos\theta, sin\theta)$ is assigned to all pixels in the image and their directional derivative is measured:

$$D_{u_\theta} f(x,y) = u_\theta{}^T \nabla f(x,y) = u_\theta{}^T \left[\frac{\partial f(x,y)}{\partial x}, \frac{\partial f(x,y)}{\partial y}\right]$$

Where $f(x,y)$ is the local grey value and $\nabla f(x,y)$ its corresponding gradient in x and y. The algorithm then finds the direction u_φ, where the derivative is maximized over the region of interest (ROI):

$$u_\varphi = \arg\max \left\| D_{u_\theta} f(x,y) \right\|_w^2 \quad , \quad \|u_\theta\| = 1$$

where $w(x,y) \geq 0$ is related with the function that describes the ROI. Thus, the magnitude of each vector is proportional to the image contrast in a given direction and the average local direction is given by a weighted sum of all the vectors. A Gaussian filter, with a variance σ, governs the effective region of interest in which the orientation should be projected, i.e. the approximate dimension (in pixels) of the local structures in the image. A range of different σ was tested to find the best orientation field that could describe our data. This parameter was settled as 4 pixels for the analysis.

From a standard inner-product manipulation we get:

$$\left\| D_{u_\theta} f(x,y) \right\|_w^2 = \langle u_\theta{}^T \nabla f(x,y), \nabla f(x,y)^T u_\theta \rangle_w = u_\theta{}^T \, \widehat{\mathrm{P}} \, u_\theta$$

Where the first eigenvector of the so-called structure tensor matrix, $\widehat{\mathrm{P}} = \langle \nabla f(x,y), \nabla f(x,y)^T \rangle$, defines the local dominant orientation. The final output is a two-dimensional orientation field, $\varphi(x,y)$, where each pixel has been replaced by unit vectors.

To estimate the curvature (k) of the orientation field, we used the definition from ref. (2), where it is described as the rate of change in the local orientation in the direction perpendicular to that orientation:

$$k(x,y) = \frac{\partial \varphi(x,y)}{\partial c} = -\sin\varphi(x,y)\frac{\partial \varphi(x,y)}{\partial x} + \cos\varphi(x,y)\frac{\partial \varphi(x,y)}{\partial y}$$

Where c corresponds to the axis perpendicular to the orientation. From this, we generated a color map (white → red → purple) containing $k\,[x_j\ y_i]$, which represents the magnitude of the

absolute curvature in φ at position x, y (Fig. 2B). As a result, this method identifies how much the orientation of a bundle is changing (to left or right) at every pixel using an arbitrary reference point. The degree of this 'kink' in the orientation field is depicted by the increasing intensity of the purple color on the heatmap, where white spaces correspond to straight filaments, i.e no kinks/curves are identified. The distribution of curvature values from each frame/heatmap were fitted to a single exponential and the mean curvature for each image $\langle k \rangle$ was given by the mean half-life of the decay (Fig. 2C).

To obtain a quantitative description of the spatial order of the filament network, we applied a nematic orientational correlation function, $S(r)$, to the angles in the orientation field.

This function compares the relative orientation of each angle with all the surrounding angles separated by an increasing distance r:

$$S(r) = \langle \cos\left(2\left(\varphi(r_{i,j})\right) - \varphi(r_l)\right)\rangle_{(|r_{i,j} - r_l| = r)}$$

Where $\varphi(r_{i,j})$ is the orientation angle at the position $r_{i,j}$ and $\varphi(r_l)$ corresponds to all the surrounding angles at the position (r_l), given that $|r_{i,j} - r_l| = r$. The angular brackets indicate an average over the indicated range and the presence of the '2' in the correlation function reflects the nematic symmetry of the system $[-\pi/2, \pi/2]$. As a result, we describe the average distance range over which the angles share a common orientation in each image. Each correlation curve typically goes from 1, where r is small and correlation is higher, to zero, where the correlation decreases drastically as the r increases. Accordingly, more ordered arrangements will display a higher spatial correlation at higher correlation lengths. To better visualize fluctuations in this parameter we identified a correlation length as r such that $S(r)$ = 0.5 (intersection) as a function of time.

Treadmilling Velocity Analysis

To quantify treadmilling dynamics, we have developed an automated image analysis based on differential image stacks and automated particle tracking using TrackMate (4), a particle tracking toolbox available for ImageJ. First, we applied a low pass Gaussian filter in space (0.5 pixels) and time (1.5 pixels) to all frames of a time-lapse movie and subtracted every two consecutive frames from one another using ImageJ ImageCalculator command. By subtracting the intensity of two consecutive frames, a new image is created where persistent pixels are removed and only short-term intensity changes are kept. Accordingly, non-moving objects generate zero pixel values, while high positive and negative intensity differences correspond to fluorescent material being added or removed at a given position, respectively. Thus, this procedure generates two new time-lapse movies showing directionally moving fluorescent spots, either corresponding to growth or shrinkage of filaments at a given position

(Fig. 4A,B). Next, using TrackMate (4), a particle tracking toolbox available for ImageJ, we identified and tracked fluorescent spots (i.e filament treadmilling). Moving spots were detected using the LoG (Laplacian Gaussian) detector with an estimated diameter of 0.8 µm. We discarded particles with a signal-to-noise ratio lower than 1 and with a track displacement distance smaller than 0.4 µm. To build the final trajectories we used the “Simple LAP tracker” with a “Max Linking Distance” of 0.3 µm, a “Maximal gap-closing distance” of 0.3 µm and “Max frame Gap” of 0 frames, and we only considered for analysis trajectories longer than 6sec.

Reconstructed trajectories were further analyzed using a custom python script to obtain velocity and directionality of the moving fluorescent spots. We computed the mean-square displacement (MSD) for each individually trajectory and estimated their velocity by fitting a quadratic equation to 50% of the total track duration (Fig S4C). The distribution of velocities recovered from these curves was fitted to a Gaussian function to extract the mean velocity at each condition (Fig. 4D-G). In addition, we calculated the autocorrelation of the velocity vectors, i.e, the angle of the normalized displacement vectors were compared pairwise as a function of an increasing time interval (Δt) and the correlation coefficient (V_{corr}) was given by the cosine of the angle difference. This kind of analysis provides information about directionality since random motion particles tend to show velocity vectors completely uncorrelated ($V_{corr} = 0$ for all Δt) while particles with a directed migration display highly correlated velocity vectors ($V_{corr} > 0$ for all Δt).

To analyze multiple time-lapse movies of treadmilling filaments in an automated manner, we created scripts based on ImageJ plug-ins and Python. These are easy to use, require no programming knowledge and we made them available as supplementary material on Github:

https://github.com/paulocaldas/Treadmilling-Speed-Analysis

The repository is organized into three different computational steps: (i) **extraction**, an ImageJ macro to automatically generate fluorescent speckles from time-lapse movies; (ii) **tracking**, an ImageJ macro that internally uses TrackMate for detection and tracking of fluorescence spots; (iii) **tracking_analysis**, a IPython notebook with detailed analysis of the trajectories generated by TrackMate. All these scrips can be applied for a single time-lapse movie or for multiple files at once in batch processing mode. This creates a highly time-efficient routine to identify and track thousands of speckles at once.

Supplementary Figures

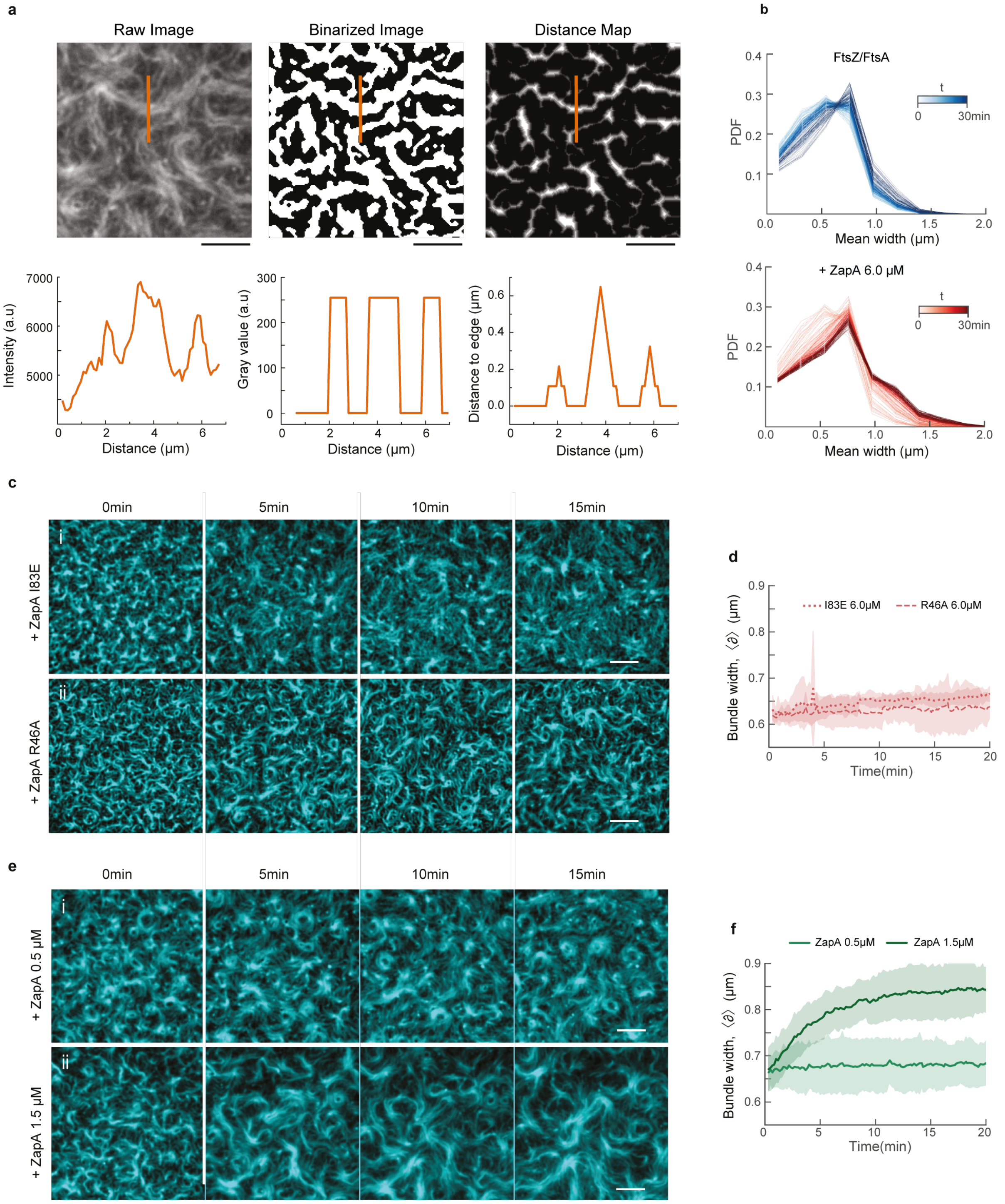

Supplementary Figure 1. Width measurements of FtsZ filament bundles

(a) To estimate bundle width, each frame in the raw data **(i)** was binarized by adaptive threshold **(ii)** before the Euclidean distance was calculated for every pixel in that frame **(iii).** Local peaks in the final gray scale image correspond to half of the bundle width (red line). Scale bars, 5µm. **(b)** The mean bundle width over time was given by the peak of widths distribution of each frame without (top, light to dark blue) and with ZapA (bottom, light to dark red).

(c) Snapshots of FtsZ pattern emerging from its interaction with FtsA and 6μM ZapA I83E **(i)** or 6μM ZapA R46A **(ii).** Scale bars, 5μm **(d)** Estimated $\langle\delta\rangle$ over time for FtsZ/FtsA filament pattern in each condition (ZapA I83E, red dotted line, n = 4; ZapA R46A, red dashed line, n = 5). **(e)** Snapshots of FtsZ pattern emerging from its interaction with FtsA and 0.5μM ZapA **(i)** or 1.5μM ZapA **(ii). (f)** Estimated $\langle\delta\rangle$ over time for FtsZ/FtsA filament pattern in each condition (ZapA 0.5 μM, green, n = 4; 1.5 μM, dark green, n = 11). Curves depict the mean and standard deviation (shade error bands) of independent experiments. Source data are provided as a Source Data file.

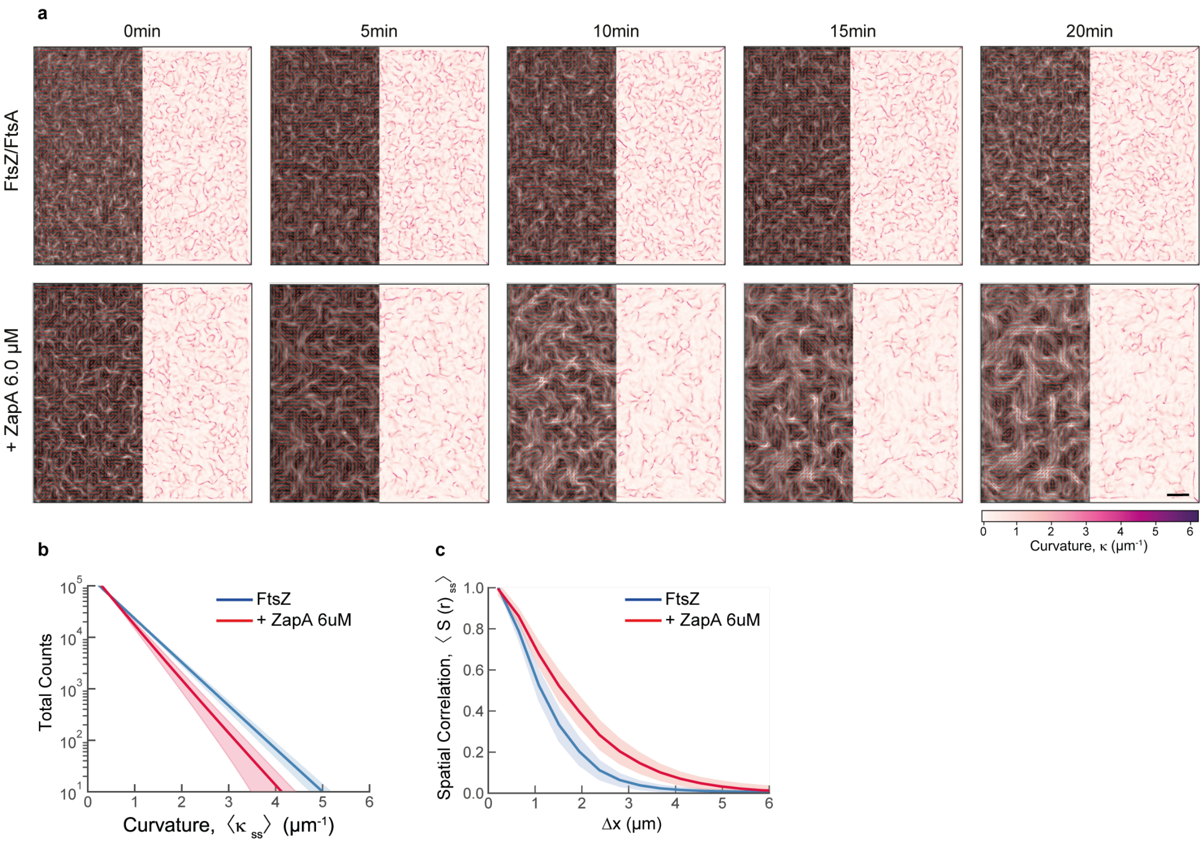

Supplementary Figure 2. Spatial order and curvature of FtsZ filament network

(a) Orientation fields and corresponding curvature maps for FtsZ filament network without (upper panel) or with 6.0 μM ZapA (lower panel) over time. Scale bars, 5 μm. **(b)** Mean curvature,$\langle k_{ss}\rangle$, and **(c)** Spatial correlation, $\langle S(r)_{ss}\rangle$, at steady-state for FtsZ/FtsA filaments alone (blue) or upon addition of 6.0 μM ZapA (red). Curves depict the mean and standard deviation (shade error bands) of independent experiments (for$\langle k_{ss}\rangle$, FtsZ, n = 10, ZapA, n = 10; for $\langle S(r)_{ss}\rangle$, FtsZ, n = 6, ZapA, n = 8). Source data are provided as a Source Data file.

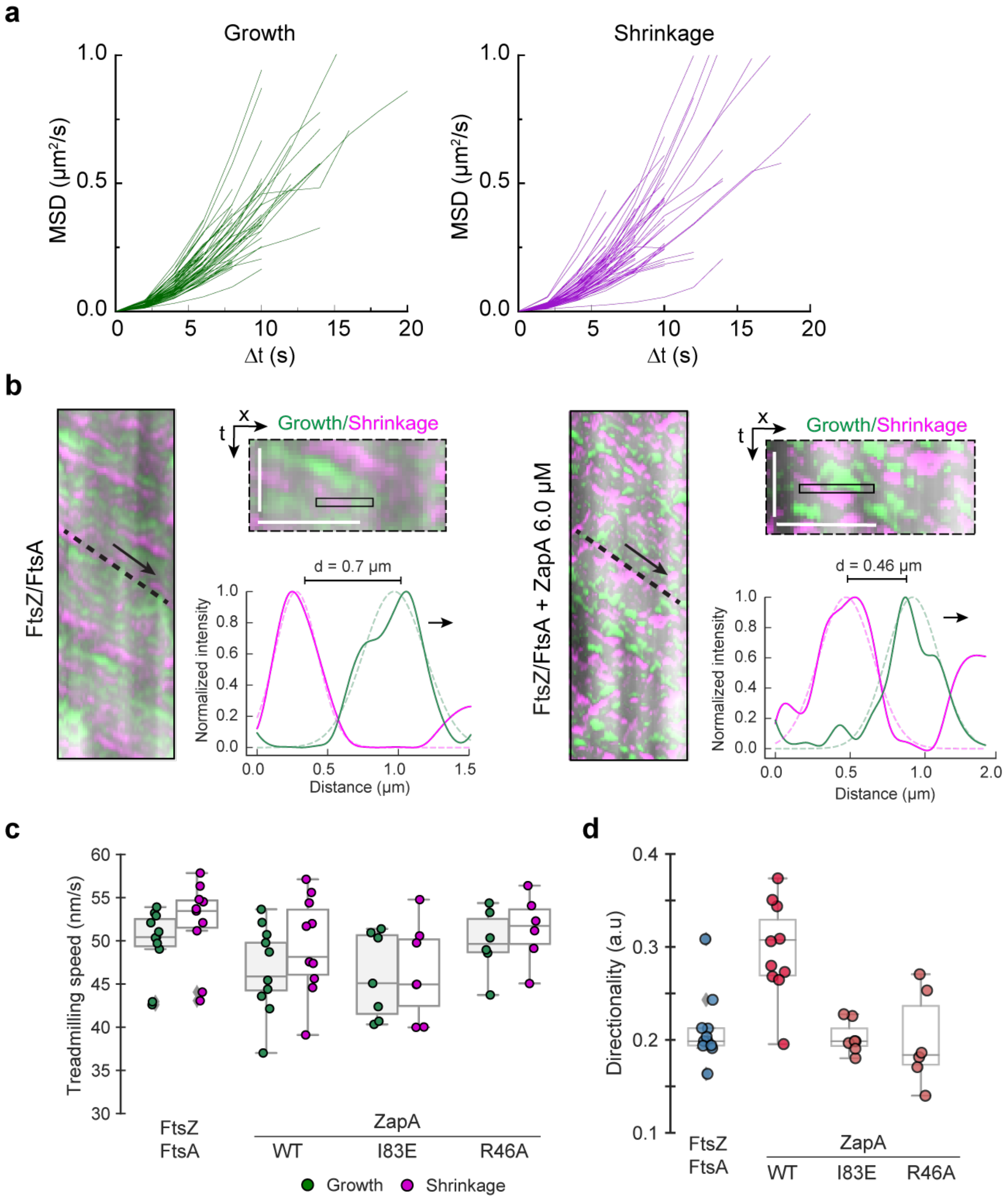

Supplementary Figure 3. ZapA does not change the orientation of filaments within bundles

(a) Representation of individual MSD curves for growth and shrinking trajectories generated from a single FtsZ/FtsA sample. **(b)** kymographs obtained along the orange line in Fig 4b for FtsZ filaments without (left panel) and in the presence of 6.0 µM ZapA (right panel). For each panel, a zoom in into the kymograph is displayed on the right and below it the respective intensity profile along the black line showing the spot intensities averaged along the treadmilling path. Arrow indicates treadmilling direction. Scale bars, t = 20 s, x = 3 µm. **(c)** ZapA mutants had no effect on treadmilling speed at either end. **(d)** Directional autocorrelation shows a significant difference for longer Δt: $\boldsymbol{\nu}_{corr}$(FtsZ) = 0.21 ± 0.04 (n=10) and $\boldsymbol{\nu}_{corr}$(ZapA) = 0.30 ± 0.05 (n=10) for Δt = 14s. Once more, ZapA mutants had no effect on this parameter ($p > 0.05$). Source data are provided as a Source Data file.

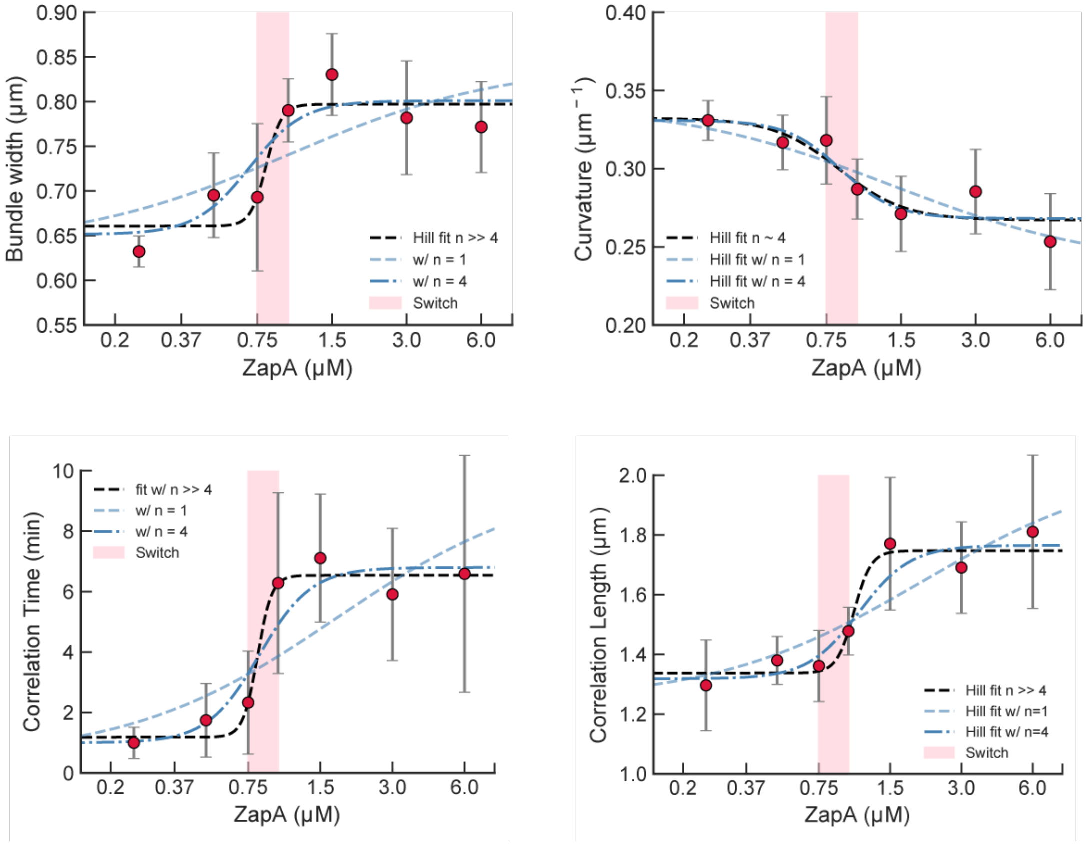

Supplementary Figure 4. Hill coefficient as measure of ultrasensitivity

Plots of Hill equation using Hill coefficients n=1, n=4 and fitting results. Data points correspond to the mean of the parameter and error bars to the standard deviation. Source data are provided as a Source Data file.

Supplementary Tables

Supplementary Table 1: Statistical significance matrix

Each matrix shows the *p value* for all the pairwise combinations of a two-tailed Student's t-tests.

Parameter		FtsZ/FtsA	ZapA WT	ZapA I83E	ZapA R46A
Bundle Width	FtsZ/FtsA	-	4.2E-03	0.13	0.080
	ZapA WT	***	-	8.6E-05	5.60E-05
	ZapA I83E	n.s	***	-	0.47
	ZapA R46A	n.s	***	n.s	-

		FtsZ/FtsA	ZapA WT	ZapA I83E	ZapA R46A
Curvature	FtsZ/FtsA	-	1.0E-06	0.01	0.437
	ZapA WT	***	-	8.5E-05	3.45E-03
	ZapA I83E	*	***	-	0.36
	ZapA R46A	n.s	***	*	-

		FtsZ/FtsA	ZapA WT	ZapA I83E	ZapA R46A
Correlation Length	FtsZ/FtsA	-	1.6E-04	0.16	0.710
	ZapA WT	***	-	2.1E-03	1.10E-04
	ZapA I83E	n.s	***	-	0.13
	ZapA R46A	n.s	***	n.s	-

		FtsZ/FtsAa	ZapA WT	ZapA I83E	ZapA R46A
Correlation Time	FtsZ/FtsA	-	2.9E-03	0.43	0.084
	ZapA WT	***	-	4.5E-03	1.32E-02
	ZapA R46A	n.s	***	-	0.23
	ZapA I83E	n.s	***	n.s	-

		FtsZ/FtsA	ZapA WT	ZapA I83E	ZapA R46A
Treadmilling Speed Growth	FtsZ/FtsA	-	0.07	0.10	0.985
	ZapA WT	n.s	-	0.28	0.34
	ZapA R46A	n.s	n.s	-	0.13
	ZapA I83E	n.s	n.s	n.s	-

		FtsZ/FtsA	ZapA WT	ZapA I83E	ZapA R46A
Treadmilling Speed Shrinkage	FtsZ/FtsA	-	0.16	0.05	0.74
	ZapA WT	n.s	-	0.73	0.13
	ZapA R46A	n.s	n.s	-	0.09
	ZapA I83E	n.s	n.s	n.s	-

		FtsZ/FtsA	ZapA WT	ZapA I83E	ZapA R46A
Directional Persistence	FtsZ/FtsA	-	6.8E-04	0.87	0.679
	ZapA WT	***	-	3.8E-04	3.95E-03
	ZapA I83E	n.s	***	-	0.73
	ZapA R46A	n.s	***	n.s	-

		FtsZ/FtsA	FtsZ + ZapA WT	FtsZ + ZapA wt
Lifetimes (SPT)	FtsZ/FtsA	-	0.470	4.4E-07
	FtsZ + ZapA WT	n.s	-	7.1E-04
	ZapA WT	***	***	-

		FtsZ/FtsA	FtsZ + ZapA WT	ZapA WT
FRAP half-time recovery	FtsZ/FtsA	-	0.140	1.4E-04
	FtsZ + ZapA WT	n.s	-	1.7E-07
	ZapA WT	***	***	-

Supplementary Table 2: Hill Equation parameter estimation from non-linear fit

Best fitted parameters for each parameter. Confidence intervals were obtained by bootstrapping the parameters obtained from non-linear regression 1000 times. Below is shown the goodness of the fit parameters for a non-linear regression fit using different hill coefficients.

	nH	c.i @ 90%	IC 50 (μM)	c.i @ 90%
Bundle Width	13.86	[13.86, 94.68]	0.81	[0.71, 0.98]
Correlation Length	12.04	[1.27, 78.81]	1.05	[0.79, 2.03]
Curvature	3.28	[1.42, 15.05]	0.84	[0.62, 1.19]
Correlation Time	14.55	[9.22, 69.27]	0.82	[0.76, 0.87]

Bundle Width

Fitting parameters	Hill equation (fitted parameters)	Hill equation with n =4 (typical cooperativity)	Hill equation with n =1 (simple graded response)
Reduced ci-squared	0.152	0.034	0.089
Bayesian info crit.	-23.4	-24.4	-16.8

Correlation Length

Fitting parameters	Hill equation (fitted parameters)	Hill equation with n =4 (typical cooperativity)	Hill equation with n =1 (simple graded response)
Reduced ci-squared	0.022	0.026	0.44
Bayesian info crit.	-27.8	-26.6	-3.92

Curvature

Fitting parameters	Hill equation (fitted parameters)	Hill equation with n =4 (typical cooperativity)	Hill equation with n =1 (simple graded response)
Reduced ci-squared	0.006	0.005	0.007
Bayesian info crit.	- 43.9	-39.4	-36.8

Correlation Time

Fitting parameters	Hill equation (fitted parameters)	Hill equation with n =4 (typical cooperativity)	Hill equation with n =1 (simple graded response)
Reduced ci-squared	0.166	0.373	1.489
Bayesian info crit.	-11.6	-5.40	5.65

Supplementary Table 3: Summary of all the parameters retrieved from the analyses

Weighted mean and respective standard deviation of each parameter for all ZapA concentrations combined, either below 0.75 μM (low ZapA) or above 1.5 μM (high ZapA). This shows how much the system changed between the two states.

		Low ZapA (< 0.5μM)			High ZapA (> 1.5μM)				
**Parameter**	**Observation**	**mean**	**s.d**	**n**	**mean**	**s.d**	**n**	**% Change**	**Figure**
Bundle width	↑	0.69	0.03	17	0.80	0.02	31	16%	Fig.1
Spatial Order	↑	1.33	0.30	13	1.76	0.05	22	33%	Fig.2
Curvature	↓	0.33	0.01	17	0.27	0.01	25	18%	Fig.2
Persistence	↑	1.27	0.30	14	6.61	0.39	25	420%	Fig.3
Growth Rate	=	50.6	2.3	17	44.1	2.53	32	n.s	Fig.5
Shrinkage Rate	=	53.1	1.9	17	46.6	2.67	32	n.s	Fig.5
Directionality	↑	0.21	0.04	10	0.30	0.05	10	41%	Fig.5
Lifetime	=	6.46	0.37	5	6.01	0.01	5	n.s	Fig.6
FRAP Half-Time	=	7.63	1.30	6	6.66	0.64	7	n.s	Fig.7

Supplementary Table 4: FtsZ/FtsA pattern quantification using with WT or Cy5-labelled ZapA

mean and respective std of each parameter in the presence of 6μM WT or Cy5-labelled ZapA

FtsZ Parameter	+ 6μM WT ZapA	+ 6μM ZapA-Cy5	t-test pval
Bundle Width (μm)	0.77 ± 0.05 (n=10)	0.85 ± 0.06 (n=3)	0.065
Correlation length (μm)	1.81 ± 0.26 (n=8)	1.62 ± 0.09 (n=4)	0.081
Curvature (μm^{-1})	0.25 ± 0.03 (n=10)	0.28 ± 0.01 (n=4)	0.027
Correlation Time (min)	6.59 ± 3.92 (n=9)	7.99 ± 4.82 (n=3)	0.551

Supplementary References

1. Theile CS, et al. (2013) Site-specific N-terminal labeling of proteins using sortase-mediated reactions. *Nat Protoc* 8(9):1800–7.
2. van Ginkel M, van de Weijer J, van Vliet LJ, Verbeek PW (1999) Curvature estimation from orientation fields. *Proc. Scandinavian Conference on Image Analysis (SCIA)*, pp 545–551.
3. Van Vliet L, Verbeek P (1995) Estimators for orientation and anisotropy in digitized images. *Proc ASCI*:442–450.
4. Tinevez JY, et al. (2016) TrackMate: An open and extensible platform for single-particle tracking. *Methods* 115:80–90.
