# Supplementary material for: Cooperative ordering of treadmilling filaments in cytoskeletal networks of FtsZ and its crosslinker ZapA
Source: Nat Commun. 2019 Dec 17;10:5744. doi: 10.1038/s41467-019-13702-4 (PMC6917738; doi:10.1038/s41467-019-13702-4)
Supplement: Supplementary file 4 — Description of Additional Supplementary Files [file 41467_2019_13702_MOESM4_ESM.docx]

**Description of Additional Supplementary Files**

File name: Supplementary Movie 1

Description: FtsZ pattern emerging from its interaction with FtsA alone (left) and in the presence of 6μM ZapA (right). See Fig. 1 for more details.

File name: Supplementary Movie 2

Description: Differential imaging allows to visualize and track FtsZ polymerization dynamics. Time-lapse movie of raw image (gray) overlaid with fluorescent spots corresponding to growing (green) and shrinking (magenta) ends of FtsZ/FtsA filament bundles. See Fig. 4 and Fig. 5 for more details.

File name: Supplementary Movie 3

Description: Time-lapse movie of raw image (gray) overlaid with fluorescent spots corresponding to growing (green) and shrinking (magenta) ends of FtsZ/FtsA filament bundles in the presence of 6µM ZapA. See Fig. 4 and Fig. 5 for more details.

File name: Supplementary Movie 4

Description: FRAP experiments on FtsZ without (left) and with (right) 6μM ZapA. See Fig. 6 for more details.

File name: Supplementary Movie 5

Description: Singe-molecule experiments on FtsZ without (left) and with (right) ZapA. See Fig. 6 for more details.

File name: Supplementary Movie 6

Description: Dual-color TIRF time lapse movie co-localization of Alexa488-FtsZ (cyan) and ZapA-Cy5 (magenta). See Fig. 7 for more details.

File name: Supplementary Movie 7

Description: Time-lapse movie of raw image (gray) overlaid with fluorescent spots corresponding to growing (cyan) and shrinking (magenta) ends of ZapA on FtsZ filament bundles. See Fig. 7 for more details

File name: Supplementary Movie 8

Description: FRAP experiments on Cy5-labelled ZapA (right). See Fig. 7 for more details.

File name: Supplementary Movie 9

Description: Singe-molecule experiments on Cy5-labelled ZapA. See Fig. 7 for more details.
