# Supplementary material for: Cooperative ordering of treadmilling filaments in cytoskeletal networks of FtsZ and its crosslinker ZapA
Source: Nat Commun. 2019 Dec 17;10:5744. doi: 10.1038/s41467-019-13702-4 (PMC6917738; doi:10.1038/s41467-019-13702-4)
Supplement: Supplementary file 2 — Peer Review [file 41467_2019_13702_MOESM2_ESM.pdf]

Reviewers' comments:

Reviewer #1 (Remarks to the Author):

The manuscript by Caldas et al., which reads very well, aims at understanding the impact of the FtsZ-associated protein ZapA on the structural and dynamic organization of the polymers of FtsZ, the main element of the bacterial division machinery in most bacteria. To achieve this aim, the authors used a very elegant in vitro reconstitution approach in supported bilayers, combined with front-line quantitative single-molecule imagining technologies. This experimental approach has previously been successfully used by the group of Martin Loose to establish the treadmilling behavior of FtsZ polymers in the presence of FtsA. The latter result was very influential in the field. In this new study, the authors have made a significant effort to improve the quality of the imaging analysis. The latter is a remarkable achievement, as it sets the standards for future studies on protein assembly systems at membrane surfaces.

Regarding the effect of ZapA on the behavior of FtsZ polymers, the study shows that ZapA increases the width of membrane-bound filaments, and stabilizes FtsZ bundles, and also as no effect on the treadmilling properties of FtsZ polymers. Most of the ZapA-related effects occur within a narrow concentration range of ZapA, which is interpreted by the authors as a signature of highly cooperative effects. Besides, the author's results show that the binding of ZapA to the FtsZ polymers is very transiently. From these results, the authors propose a mechanism to explain the role of ZapA on the FtsZ polymers. This model may contribute to the precision and stability of the mode of action of the division machinery.

1) Although the quality of the writing and the experiments is very high, I have some difficulties with the claiming that ZapA acts on the FtsZ filaments in a highly cooperative manner, that will need further clarification as they are central to the main conclusions of the paper.

Pag. 6, line, 125 - the Hill coefficient (n) is 4.12 +/- 6.84. With such high uncertainty, one cannot discriminate the mode of association, as a single Langmuir isotherm (n = 1) can also explain the data within the experimental error of the best fit parameter values. The same happens with the Hill coefficient shown in pag. 8, line 199 (N = 4.76 +/- 4.21).

I would appreciate if the authors revise this analysis in other to make the claims on the mode of association. For example, I would recommend plotting all the concentration dependence data in log scale of the X-axis, which will help the readers to appreciate the nature of the processes visually.

2) The text in the Results describing that ZapA does not affect treadmilling velocity (pag 9, line 241) is the one only that it is not clear for a general reader. Please improve.

3) In the experiments showing that ZapA binds transiently to the FtsZ filaments (pag 11, line 305) uses a variant of ZapA to monitor the behavior of this protein independently of FtsZ. I would appreciate the authors adding a comment on the controls done to make sure that the variant of ZapA and the original version behaves similarly.

4) The discussion section reads well as it presents the principles, relationships, and generalizations shown by the results. However, my recommendation is to revise the whole mechanistic part, taking into account point 1 of this commentary, which I consider central for the entire study.
The comparison with other self-assembling systems, like actin and tubulin polymers, is well done. In this point, I would suggest the authors consider to complete this section by including a comment on the possibility of entropic forces driving force generation of cytoskeletal networks. In this regard, the paper by Braun et al. 2016 Bioessays 38: 474-481 might be useful.

Reviewer #2 (Remarks to the Author):

In this manuscript, the authors studied how the well-conserved protein ZapA organizes FtsZ filament structures and treadmilling dynamics using a previously established supported lipid bilayer system. The authors found that ZapA cooperatively increases the spatial order of the filament network: FtsZ bundles become thicker, straighter, and more correlated in time and space above a certain ZapA concentration. Curiously, the binding of ZapA to FtsZ is transient and does not change FtsZ's treadmilling dynamics at all. While the results did not come as a surprise because in vivo studies have shown that ZapA is non-essential and has little effect on FtsZ's treadmilling dynamics (and hence one may argue what new insight this work could bring), I strongly encourage the publication of this work because it provided the most quantitative, comprehensive characterizations of the structure and kinetics of the incredibly dynamic polymer network formed by FtsA, FtsZ and ZapA. These quantitative measurements are impossible in vivo. They pointed to a model in which ZapA can increase the precision and stability of the Z-ring, facilitate its assembly during cell division, and hence supporting the scaffolding function of the Z-ring for divisome assembly. Additionally, the analyses developed by the authors to extract meaningful biochemical measurements from the complex filament network will greatly benefit other researchers in the field.

I think the manuscript can be accepted as it is now, but do wish the authors to spend a bit more time to address how the transient interactions of ZapA with FtsZ could increase the stiffness of FtsZ filaments. I found the reasoning of liquid droplets-like weak interactions not convincing. Additionally, how do the authors reconcile that previous biochemical data showing that ZapA reduces FstZ's GTPase activity, but here in this work ZapA does not change FstZ's treadmilling dynamics, which are dependent on GTP hydrolysis? Perhaps previous work was measured in solution and here it is on supported lipid bilayers?

Reviewer #1 (Remarks to the Author):

The manuscript by Caldas et al., which reads very well, aims at understanding the impact of the FtsZ-associated protein ZapA on the structural and dynamic organization of the polymers of FtsZ, the main element of the bacterial division machinery in most bacteria. To achieve this aim, the authors used a very elegant in vitro reconstitution approach in supported bilayers, combined with front-line quantitative single-molecule imagining technologies. This experimental approach has previously been successfully used by the group of Martin Loose to establish the treadmilling behavior of FtsZ polymers in the presence of FtsA. The latter result was very influential in the field. In this new study, the authors have made a significant effort to improve the quality of the imaging analysis. The latter is a remarkable achievement, as it sets the standards for future studies on protein assembly systems at membrane surfaces.

Regarding the effect of ZapA on the behavior of FtsZ polymers, the study shows that ZapA increases the width of membrane-bound filaments, and stabilizes FtsZ bundles, and also as no effect on the treadmilling properties of FtsZ polymers. Most of the ZapA-related effects occur within a narrow concentration range of ZapA, which is interpreted by the authors as a signature of highly cooperative effects. Besides, the author's results show that the binding of ZapA to the FtsZ polymers is very transiently. From these results, the authors propose a mechanism to explain the role of ZapA on the FtsZ polymers. This model may contribute to the precision and stability of the mode of action of the division machinery.

1) Although the quality of the writing and the experiments is very high, I have some difficulties with the claiming that ZapA acts on the FtsZ filaments in a highly cooperative manner, that will need further clarification as they are central to the main conclusions of the paper.

Pag. 6, line, 125 - the Hill coefficient (n) is 4.12 +/- 6.84. With such high uncertainty, one cannot discriminate the mode of association, as a single Langmuir isotherm (n = 1) can also explain the data within the experimental error of the best fit parameter values. The same happens with the Hill coefficient shown in pag. 8, line 199 (N = 4.76 +/- 4.21).

I would appreciate if the authors revise this analysis in other to make the claims on the mode of association. For example, I would recommend plotting all the concentration dependence data in log scale of the X-axis, which will help the readers to appreciate the nature of the processes visually.

We thank the reviewer for pointing this out. First, we want to emphasize that in our study, we used the Hill coefficient merely as a global measure of cooperative behavior. The Hill coefficient we obtained does not allow to provide a molecular-level interpretation of our data.

Motivated by the reviewer's important comment, we first performed additional experiments to include another data point at a ZapA concentration of 1.0 µM, i.e. in the transition region between 0.75 µM and 1.5 µM ZapA. With this additional data point we verified the switch-like, non-linear behavior of the FtsZ-ZapA system: two different stable states and a critical ZapA concentration that triggers the transition between them. These three features are inconsistent with a graded response.

Next, following the reviewer's advice, we have improved our analysis and fitting procedure. Previously, the non-linear regression we used in our analysis relied on the assumption that the Hill coefficient relies on a normal distribution. Now, we have implemented a bootstrap method for the robust estimation of fitting parameters without underlying assumptions. After the initial fitting procedure is performed, this method generates new sets of data points by reshuffling randomly the residuals of the fit. The new data sets generated are then used to perform new fits and obtain a

distribution of fitting parameters. Using this method, we could robustly calculate the 90% confidence interval of the distribution, which consistently shows that the corresponding Hill coefficient is much larger than 1. Interestingly, we found that the distribution of values obtained for the Hill coefficient was bimodal, presumably because the range and number of concentrations used in our titration experiments was limited.

Finally, to convince the reader that fitting a Hill equation with a Hill coefficient nH > 4 provides a better fit than with nH = 1, we show the corresponding curves in the supplemental data (Supplementary Figure 4) and used two objective evaluation criteria: the reduced ci-squared values and Bayesian Info Criterium (BIC). Importantly, by calculating ΔBIC = BIC(nH=1) – BIC(nH > 4) to compare different Hill coefficients, we can objectively exclude nH = 1 as the best possible fit (see Supplementary Table 5 and Supplementary Figure 4).

We want to thank the reviewer again for these comments, as it helped use to improve our analysis and to more convincingly show that the effect of ZapA on FtsZ is highly cooperative.

2) The text in the Results describing that ZapA does not affect treadmilling velocity (pag 9, line 241) is the one only that it is not clear for a general reader. Please improve.

We thank the reviewer for pointing this out. We believe that the message of this section is now clearer (see lines 412).

3) In the experiments showing that ZapA binds transiently to the FtsZ filaments (pag 11, line 305) uses a variant of ZapA to monitor the behavior of this protein independently of FtsZ. I would appreciate the authors adding a comment on the controls done to make sure that the variant of ZapA and the original version behaves similarly.

We thank the reviewer for this comment since confirming the activity of the fluorescently labeled protein is a very important control. Indeed, we had not provided a quantitative comparison for the effect of non-labelled and Cy5-labelled ZapA on the FtsZ/FtsA pattern.

We provide now an additional table (**Supplementary Table 4 and below)** containing a quantitative description of the architecture and reorganization dynamics of the FtsZ filament network in the presence of 6µM ZapA-Cy5, as well as a comparison with the values obtained for 6µM non-labeled ZapA (WT ZapA). As can be seen from these results, C-terminal labelling of ZapA with Cy5 does not affect its activity on FtsZ filaments in our experiments.

**FtsZ Parameter**	**+ 6µM WT ZapA**	**+ 6µM ZapA-Cy5**	**t-test pval**
Bundle Width (µm)	0.76 ± 0.05 (n = 13)	0.85 ± 0.06 (n=3)	0.065
Correlation length (µm)	1.74 ± 0.21 (n = 8)	1.62 ± 0.09 (n=4)	0.081
Curvature (μm^{-1})	0.26 ± 0.03 (n = 8)	0.28 ± 0.01 (n=4)	0.027
Correlation Time (min)	5.96 ± 3.79 (n =14)	7.99 ± 4.82 (n=3)	0.551

Supplementary Table 4: FtsZ/FtsA pattern quantification with WT or Cy5-labelled ZapA. Mean and respective standard deviation for each parameter obtained in our analyses in the presence of 6µM WT or Cy5-labelled ZapA.

4) The discussion section reads well as it presents the principles, relationships, and generalizations shown by the results. However, my recommendation is to revise the whole mechanistic part, taking into account point 1 of this commentary, which I consider central for the entire study.

The comparison with other self-assembling systems, like actin and tubulin polymers, is well done. In this point, I would suggest the authors consider to complete this section by including a comment on the possibility of entropic forces driving force generation of cytoskeletal networks. In this regard, the paper by Braun et al. 2016 Bioessays 38: 474-481 might be useful.

We thank the reviewer for his positive comments and bringing the very interesting paper by the Diez and Dogic lab to our attention. We read this review as well as the corresponding research paper by Lansky *et al.*[1] carefully and can conclude that the biggest difference between the ZapA-FtsZ and Ase1-microtubule system is that Ase1, in contrast to ZapA, shows a very long residence time on the filament lattice. This persistent binding time allows Ase1 to diffuse along the microtubules lattices, and to generate entropic forces. In contrast, the residence time of ZapA is simply too short for such kind of effect on FtsZ filaments. We think this is a very important difference between these two proteins systems and therefore include it in our discussion (lines 489 ff.).

<u>Reviewer #2 (Remarks to the Author):</u>

In this manuscript, the authors studied how the well-conserved protein ZapA organizes FtsZ filament structures and treadmilling dynamics using a previously established supported lipid bilayer system. The authors found that ZapA cooperatively increases the spatial order of the filament network: FtsZ bundles become thicker, straighter, and more correlated in time and space above a certain ZapA concentration. Curiously, the binding of ZapA to FtsZ is transient and does not change FtsZ's treadmilling dynamics at all. While the results did not come as a surprise because in vivo studies have shown that ZapA is non-essential and has little effect on FtsZ's treadmilling dynamics (and hence one may argue what new insight this work could bring), I strongly encourage the publication of this work because it provided the most quantitative, comprehensive characterizations of the structure and kinetics of the incredibly dynamic polymer network formed by FtsA, FtsZ and ZapA. These quantitative measurements are impossible in vivo. They pointed to a model in which ZapA can increase the precision and stability of the Z-ring, facilitate its assembly during cell division, and hence supporting the scaffolding function of the Z-ring for divisome assembly. Additionally, the analyses developed by the authors to extract meaningful biochemical measurements from the complex filament network will greatly benefit other researchers in the field.

I think the manuscript can be accepted as it is now, but do wish the authors to spend a bit more time to address how the transient interactions of ZapA with FtsZ could increase the stiffness of FtsZ filaments. I found the reasoning of liquid droplets-like weak interactions not convincing. Additionally, how do the authors reconcile that previous biochemical data showing that ZapA reduces FstZ's GTPase activity, but here in this work ZapA does not change FstZ's treadmilling dynamics, which are dependent on GTP hydrolysis? Perhaps previous work was measured in solution and here it is on supported lipid bilayers?

We want to thank the reviewer for such positive feedback on our work! The reviewer is correct that previous studies found a decreased GTPase activity of FtsZ in the presence of ZapA and that this effect would imply decreased treadmilling dynamics. In contrast, we found that ZapA has no effect on the treadmilling velocity. We want to point out that a reduction of GTPase activity was only found at a non-physiological pH of 6.5, which promotes intrinsic bundling of FtsZ[2–4]. In contrast, the effect

was almost absent at a physiological pH of 7.5 (ref. 5). Furthermore, as the reviewer pointed out correctly, the potential effect of the membrane on the GTPase activity was not addressed in these previous studies. In fact, these conflicting reports were one of the reasons why it was important to find out how ZapA actually affects the treadmilling behavior of membrane-bound FtsZ (see lines 49-51 and 280-288). Our results now unambiguously show that ZapA has no effect on the polymerization dynamics of membrane-bound FtsZ filaments, while it changes the architecture of the filament network.

How ZapA can increase the stiffness of FtsZ filaments is an interesting point. We believe that the reviewer is referring to the study by Dajkovic *et al*., who used rheometry measurements to quantify the elastic modules of FtsZ gels *in vitro*[6]. This study showed that intrinsic bundling of FtsZ filaments (facilitated by the presence of 10 mM $CaCl_2$ or of a truncated version of FtsZ) does not increase the stiffness of a FtsZ filament network, while the presence of ZapA does. It is obvious from our experiments that despite the transient nature of an individual ZapA-FtsZ interaction, ZapA strongly influences the alignment of FtsZ filaments and as a consequence the large-scale the architecture of the filament network. Furthermore, from experiments on actin filament networks, it is known that even transient binding of crosslinking proteins effects the mechanical properties of the actin gel. Accordingly, transient binding of ZapA does not contradict an increase stiffness of FtsZ filaments. We now refer to the increase of filament bundle stiffness and the study by Dajkovic *et al*. in our discussion (line 360).

References

1. Lansky, Z. *et al.* Diffusible crosslinkers generate directed forces in microtubule networks. *Cell* (2015). doi:10.1016/j.cell.2015.01.051

2. Small, E. *et al.* FtsZ Polymer-bundling by the Escherichia coli ZapA Orthologue, YgfE, Involves a Conformational Change in Bound GTP. *J. Mol. Biol.* **369**, 210–221 (2007).

3. Galli, E. & Gerdes, K. FtsZ-ZapA-ZapB interactome of Escherichia coli. *J. Bacteriol.* **194**, 292–302 (2012).

4. Pacheco-Gómez, R. *et al.* Tetramerization of ZapA is required for FtsZ bundling. *Biochem J* **449**, 795–802 (2013).

5. Mohammadi, T. *et al.* The GTPase activity of Escherichia coli FtsZ determines the magnitude of the FtsZ polymer bundling by ZapA in vitro. *Biochemistry* **48**, 11056–11066 (2009).

6. Dajkovic, A., Pichoff, S., Lutkenhaus, J. & Wirtz, D. Cross-linking FtsZ polymers into coherent Z rings. *Mol Microbiol* **78**, 651–668 (2010).

REVIEWERS' COMMENTS:

Reviewer #1 (Remarks to the Author):

The authors have addressed all of my concerns. The revised version of the manuscript has considerably improved. The paper deserves publication in Nature Communications.

Reviewer #2 (Remarks to the Author):

The authors have addressed all my concerns. I support its acceptance by Nat Communications.

REVIEWERS COMMENTS:

Reviewer #1 (Remarks to the Author):

The authors have addressed all of my concerns. The revised version of the manuscript has considerably improved. The paper deserves publication in Nature Communications.

Reviewer #2 (Remarks to the Author):

The authors have addressed all my concerns. I support its acceptance by Nat Communications.

Thanks a lot! We want to thank the reviewers for the helpful reports and are very happy that we were able to clarify their concerns.
